# Traditional zootherapeutic studies in India: a review

**DOI:** 10.1186/1746-4269-4-17

**Published:** 2008-07-18

**Authors:** Madan Mohan Mahawar, DP Jaroli

**Affiliations:** 1 Department of Zoology, Government Post Graduate College, Sawai Madhopur, Rajasthan, India; 2 Department of Zoology, University of Rajasthan, Jaipur, Rajasthan, India

## Abstract

The present study aims to review the zootherapeutic practices of the different ethnic communities of India. This work is also an attempt to present a list of animals' use for medicinal purposes by different communities of India. Data were gathered from 15 published research papers of various authors on zootherapeutic studies in India from 2000 to 2007. Approximately 109 animals and their 270 uses are reported in traditional medicine in different parts of India. Of these, the highest numbers of animal species (42, 38.5%) with 50 (18.5%) uses have been reported for the treatment of Respiratory system related problems. Rheumatic and other pains are treated with 32 species (29.4%) in 34 (12.9%) uses. Gastric problems are reported to be treated with 22 (20.2%) species in 26 (9.9%) uses. The mammals constitute the highest number of animals used for medicinal purposes. 44 (40%) mammals, 24 (22%) invertebrates, 18 (17%) birds, 12 (11%) reptiles, nine (8%) fishes and two (2%) amphibians have been reported for medicinal purposes. Of the total 109 animal species reported, 76(70%) are included in IUCN red data list and 36 (33%) animal species are listed in CITES appendix I, II, and III. This work will be helpful in biodiversity conservation in India and also give a clue to investigate bio-active compound in these animal raw materials.

## Background

The world health organization estimates that as many as 80% of the world's more then six billion people rely primarily on animal and plant based medicine [1]. In modern societies, zootherapy constitutes an important alternative among many other known therapies practiced worldwide. Wild and domestic animals and their byproducts (e.g. hooves, skin, bones, feathers and tusks) form important ingredients in the preparation of curative, protective and preventive medicine [2]. For example, in Traditional Chinese Medicine (TCM) more then 1500 animal species have been recorded to be some medicinal use [3]. Of the 252 essential chemicals that have been selected by the World Health Organization, 11.1% come from plants, and 8.7% from animals [4]. And of the 150 prescription drugs currently in use in the United States of America, 27 have animal origin [5]. In India nearly 15–20 percent of the Ayurvedic medicine is based on animal-derive substance [6]. In Unani system of medicines about 200 drug of animal origin are described which are claimed to be beneficial for the treatment of the various ailments [7]. In Bahia state, in the northeast of Brazil, over 180 medicinal animals have been recorded [8]. In Pakistan 31 substances were listed (animal parts and products), constituting 9% of all the medicinal substances in the inventory of traditional medicines [9].

In India, since times immemorial, great work was done in this field and documented in works like *Ayurveda *and *charaka Samhita*. Additionally immense knowledge has come down to modern times through folklore as various practices became a part of tradition amongst various groups. We can find that people still use various animal products and by-products for cure of various diseases. For example, honey is used as expectorant, cattle urine has been used as a therapeutic. All this knowledge has once again come to the limelight, as there has been a sort of disillusionment with the current allopathic cure, as it has got its own side effect and in fact has no cure for various diseases. Therefore people are looking for traditional remedies for the treatment of ailments. But in India this traditional knowledge is fast eroding due to modernization. Thus there is an urgent need to inventorise and record all ethnobiological information among the different ethnic communities before the traditional cultures are completely lost [10]. Therefore, ethnobiologist have a greater responsibility not only in inventorising the traditionally used biological resources but also in conserving and revitalizing the traditionally beliefs, so that the age-old cultures are not lost. The studies on the therapeutic uses of animals and their parts have been neglected, when compare to plants [11]. Thus there is an urgent need to make such study in the field of ethnozoology and document it, so that it can be put to the welfare of human kind.

Many ethnobiologist are collected zootherapeutic information from different ethnic groups or tribes in India. S.K. Sharma describes use of animals to cure ailments of human beings and domestic cattle by Bhil tribe of Rajasthan. [12]. Jamir and Lal describe the traditional method of treating various kinds of ailments using twenty six animal species and their products by different Naga tribes [13]. Patil found that the tribals of Nandurbar district (Maharashtra) have been use wild animal parts as medicines along with plants. This study assesses 15 species of animals used by the tribals like Bhils, Gamits, Koknas and Pawaras as medicine [14]. Ranjit Singh et al describe the Ethno-entomological practices in Tirunelveli district, Tamil Nadu. In this investigation, 11 species of insects used to prepare traditional medicine [15]. Banerjee et al describe that Honey, as a product from bees, has multiple properties, and is being therapeutically used since time immemorial. It's antibacterial, anti-inflammatory and wound healing properties are promising [16]. Gupta et al describe the traditional knowledge of local communities in district Kachchh and listed about 34 animal species, which are used in primary health care needs of human beings and livestock [17]. Kalita et al study the plant and animal based folk medicine used by people of Dibrugarh district, Assam for treatment of eleven different diseases. In this study, information on utility of 19 plant species and four animal species is collected [18]. Solavan A et al carried out a study among nine tribes spread over four districts of Tamil Nadu, India and identified the traditional therapeutic uses of sixteen different animal's species, consisting of six mammals, five birds, two reptiles, two arthropod and one annelid for the treatment of over 17 kinds of ailments [10]. Mahawar and Jaroli carried out a study among the inhabitants, whose are living surrounding the Ranthambhore National Park, India and identified a total of 15 animals were used comprising 20 therapeutic purposes [19]. Mahawar and Jaroli [2007] carried out a study among the Saharia tribe and identified a total of 15 animal species were recorded and they are used for different ethnomedical purposes, including cough, asthma, tuberculosis, paralysis, earache, herpes, weakness, muscular pain, etc [20]. The Chakhesang tribe of Nagaland also uses twelve mammals, one bird, one reptile, two amphibians, one fish, one mollusk, one annelid and four arthropods for treatment of various ailments [21]. Kakati and Doulo studied Ao tribe of Nagaland and identified twenty five different vertebrate species for traditional therapeutic use, of which, some have become rare [22]. Oudhia describe three animal's medicinal uses, which are reported by traditional healers and natives of Bhopalpatnam region, Chhattisgarh, India. These native have rich traditional medicinal knowledge about common herbs insects and other animals [23]. Oudhia also describe the traditional Medicinal knowledge about excreta of ten animals used to treat many common diseases in Chhattisgarh, India [24]. Insects, mites, and spiders are used as medicines to cure both common and complicated ailments in Chhattisgarh, India. For example, the oil from the red velvet mite (*Trombidium grandissimum (Koch, 1867) *is useful for paralysis. Also due to its ability to increase the sexual desire, these mites are named as Indian Viagra [25].

This study deals to summarized and review on the zootherapeutic practices by the different ethnic communities of India. This work is also an attempt to present a list of animal's uses for medicinal purposes by different communities of India. The authors hope that this work will be helpful in biodiversity conservation in India and also give a clue to investigate bio-active compound in these animal raw materials.

## Methods

Data were gathered from 15 published research papers of various authors on zootherapeutic studies in India from 2000 to 2007 (Table 1). The majority of these papers contain English name, scientific name, area or tribe reported, part or product or raw material name and mode of preparation, etc. All the medicinal uses of animals are classified in 14 major disease categories i.e. Antidote, Burn, Eye and Ear, Gastric disorder, Gynecological problems, Impotency, Nervous System, Pains, Respiratory Problem, Skin related Problem, Urinary Problem, Weakness and Wound healing. These categories are forms to show all related health problems in a major group. For example asthma, cough, cold, tuberculosis or any other respiratory problems are presented into a major disease category called respiratory system related problems.

**Table 1:** List of published research works on Ethnomedicinal uses of animals in different parts of India.

Tribes/Ethnic Groups/Region/ Indigenous people	Number of animals reported	Authors
Chakhesang of Nagaland	23	Kakati and Doulo (2000)
Bhil of Rajasthan	17	Sharma S K (2002)
Bhil, Gamit, Kokna, etc of Maharastra	15	Patil S H (2003)
Bhopalpatnam (chhattisgarh)	3	Oudhia P (2003a)
Chhattisgarh	10	Oudhia P (2003b)
Chhattisgarh	7	Oudhia P (2005)
Kachch (Gujrat)	34	Gupta Leena et al (2003)
Irular, Kurimba of Tamilnadu	26	Solvan A et al (2004)
Kanikar, Paliyar of Taminadu	11	Ranjit Singh ASA (2004)
Naga tribe of Nagaland	26	Jamir N S et al (2005)
Dibrugarh (Assam)	4	Dilip Kalita (2005)
Ao tribe of Nagaland	25	Kakati L N et al (2006)
Mogya, Meena, Bawaria of Rajasthan	15	Mahawar, Jaroli (2006)
Shoka tribe of Uttaranchal	36	Negi and palyal (2007)
Saharia of Rajasthan	15	Mahawar, Jaroli (2007)

We summarized all the medicinal information in 14 disease categories table. Each disease category table contains information in the following pattern: English name, scientific name, area or tribe reported, part or product or raw material name, mode of preparation and reference of the authors (additional file 1).

The valid scientific names with author's names of the animal's species were included in the database. Many times authors have given synonyms of animal species in their publications. These data are updated according to the ITIS Catalogue of Life, Annual Checklist (2007) and NCL Centre for Biodiversity Informatics (NCBI) [26,27] (Table 2). The conservation status of the animal species follows IUCN (2007) and CITES (2007) [28,29].

**Table 2:** List of animals uses as medicinal purposes in different parts of India.

S. N.	Category	*Scientific name*	English name	Red data list	CITES
1.	Invertebrate	*Apis cerana indica – (Fabricius 1798)*	Honey bee		
2.	Invertebrate	*Apis dorsata (Fabricius, 1793)*	(Rock bee)		
3.	Invertebrate	*Apis florea (Fabricius, 1787)*	(Little bee)		
4.	Invertebrate	*Blatta orientalis Linnaeus, 1758 – valid – blatte orientale, oriental cockroach*	Cockroach		
5.	Invertebrate	*Bombyx mori (Linnaeus)*	Silkworm		
6.	Invertebrate	*Cancer pagurus (Linnaeus, 1758)*	Crab		
7.	Invertebrate	*Cimex lectularius (Linnaeus, 1758)*	Bed Bug		
8.	Invertebrate	*Cimex rotundatus (Signoret, 1852)*	Bed Bug		
9.	Invertebrate	*Dactylopius coccus (Costa, 1835)*	Cochineal insect		
10.	Invertebrate	*Dasymutilla occidentalis (Linnaeus)*	Velvet ant		
11.	Invertebrate	*Dorylus labiatus Shuckard, 1840*	Ant		
12.	Invertebrate	*Helicoverpa armigera (Hübner, 1805)*	Pod Borer		
13.	Invertebrate	*Heterometrus swammerdami (Simon, 1872) Synonym – Palamnaeus swammerdami*	Scorpion		
14.	Invertebrate	*Kerria lacca (Kerr, 1782)*	Lac insect		
15.	Invertebrate	*Macrobrachium malcolmsonii (H. Milne-Edwards, 1844)*	Prawn		
16.	Invertebrate	*Matuta planipes (Fabricius, 1798) Synonym-Matuta victor*	Sandy shore Crab		
17.	Invertebrate	*Musca domestica nebulo (Fabricius. 1784)*	Housefly		
18.	Invertebrate	*Nephotettix nigropictus (Stal), 1870*	Green Leafhopper (GLH)		
19.	Invertebrate	*Oecophylla smaragdina (Fabricius, 1775)*	Weaver ant		
20.	Invertebrate	*Pheretima posthuma (L. Vaillant) 1868*	Earthworm		
21.	Invertebrate	*Photuris lucicrescens (Barber, 1951)*	Lightening Beetles or Fireflies or Lighting bugs		
22.	Invertebrate	*Pila globosa (Swainson, 1822)*	Apple Snail		
23.	Invertebrate	*Trombidium grandissimum (Koch, 1867)*	Red Velvet Mite		
24.	Invertebrate	*Uca pugnax*	Hermit Crab		
25.	Pisces	*Amphipnous cuchia (Hamilton, 1822).*	Eel		
26.	Pisces	*Monopterus cuchia (Hamilton, 1822)*	cuchia eel		
27.	Pisces	*Schizothorax richardsonii (Gray, 1832)*	Fish		
28.	Pisces	*Monopterus albus (Zuiew, 1793)*	Eel Fish	Data deficient	
29.	Pisces	*Tor putitora (Hamilton, 1822)*	Fish	Endangered	
*30.*	Pisces	*Channa punctata (Bloch, 1793) Synonym-Channa punctatus Linn.*	*Channa*	Least concern	
31.	Pisces	*Labeo gonius (Hamilton, 1822)*	carp fish	Least concern	
32.	Pisces	*Labeo rohita (Hamilton, 1822)*	Labeo	Least concern	
33.	Pisces	*Eusphyra blochii (Cuvier, 1816) *Synonym-Zygaena blochii	Hammer head shark	Near threatened	
34.	Amphibian	*Fejervarya limnocharis *synonym-*Lymnonecties limnorcharis*	Frog	Vulnerable	
35.	Amphibian	*Hoplobatrachus tigerinus (Daudin, 1803) synonym-Rana tigrina*	Frog	Vulnerable	II
36.	Reptile	*Gloydius himalayanus (Günther, 1864) *Synonym-*Ancistrodon himalayans*	Snakes	Data Deficient	
37.	Reptile	*Eryx johnii (Russell, 1801)*	Earth Boa	Least concern	II
38.	Reptile	*Naja naja (Linnaeus, 1758)*	Cobra	Near threatened	II
39.	Reptile	*Calotes versicolor (Fitzinger, 1826)*	Common Garden Lizard	Near threatened	
40.	Reptile	*Lissemys punctata (Lacépède, 1788)*	Indian Flap shell turtle	Near threatened	II
41.	Reptile	*Ptyas mucosus (Linnaeus, 1758)*	Snakes	Near threatened	II
42.	Reptile	*Python reticulatus (Schneider, 1801)*	python	Near threatened	II
43.	Reptile	*Daboia russelii (Shaw & Nodder, 1797) *Synonym-*Vipera russelli*	Snakes	Near threatened	III
44.	Reptile	*Varanus bengalensis (Daudin, 1758)*	Monitor	Vulnerable	I
45.	Reptile	*Kachuga tentoria (Gray, 1834)*	Hard shelled Turtle.	Vulnerable	II
46.	Reptile	*Uromastyx hardwickii (Gray, 1827)*	Spiny tailed lizard	Vulnerable	II
47.	Reptile	*Varanus salvator (Laurenti, 1768)*	Monitor	Vulnerable	II
48.	Aves	*Acridotheres ginginianus (Latham, 1790)*	Bank myna	Least concern	
49.	Aves	*Centropus sinensis (Stephens, 1815)*	Crow-pheasant	Least concern	
50.	Aves	*Coracias benghalensis (Linnaeus, 1758)*	Indian Roller	Least concern	
51.	Aves	*Corvus splendens (Vieillot, 1817)*	Crow	Least concern	
52.	Aves	*Gallus gallus domesticus*	hen	Least concern	
53.	Aves	*Halcyon smyrnensis (Linnaeus, 1758)*	White-breasted Kingfisher	Least concern	
54.	Aves	*Passer domesticus (Linnaeus, 1758)*	House sparrow	Least concern	
55.	Aves	*Pavo cristatus (Linnaeus, 1758)*	Indian Peafowl	Least concern	
56.	Aves	*Pseudibis papillosa (Temminck, 1824)*	Black ibis	Least concern	
57.	Aves	*Streptopelia decaocto (Frivaldszky, 1838)*	Collared dove	Least concern	
58.	Aves	*Vanellus indicus (Boddaert, 1783)*	Red-wattled bird	Least concern	
59.	Aves	*Aquila rapax (Temminck, 1828)*	Eagle	Least concern	II
60.	Aves	*Gallus sonneratii (Temminck, 1813)*	Jungle fowl	Least concern	II
61.	Aves	*Strix aluco nivicolum (Blyth, 1845)*	Owl	Least concern	II
62.	Aves	*Tyto alba (Scopoli, 1769)*	Barn or Screech Owl	Least concern	II
63.	Aves	*Columba livia (Gmelin, 1789)*	Pigeon	Least concern	III
64.	Aves	*Martes flavigula (Boddaert, 1785)*	Martens bird	Least concern	III
65.	Aves	*Catreus wallichii (Hardwicke, 1827)*	Chir pheasant	Vulnerable	I
66.	Mammal	*Myotis lucifugus (LeConte, 1831)*	Bat	Conservation Dependent	
67.	Mammal	*Bison bison (Linnaeus, 1758)*	Bison	Conservation Dependent	II
68.	Mammal	*Equus asinus (Linnaeus, 1758)*	Donkey Indian		
69.	Mammal	*Panthera tigris (Linnaeus, 1758)*	Tiger	Endangered	I
70.	Mammal	*Bubalus bubalis (B. arnee) (Linnaeus, 1758)*	Buffalo		
71.	Mammal	*Capra falconeri (Wagner, 1839)*	goat	Endangered	I
72.	Mammal	*Camelus dromedarius (Linnaeus, 1758)*	Camel	Least concern	
73.	Mammal	*Capra sibirica (Pallas, 1776)*	goat	Least concern	
74.	Mammal	*Cervus unicolor (Kerr, 1792)*	Sambhar	Least concern	
75.	Mammal	*Cynopterus sphinx (Vahl, 1797)*	Bat	Least concern	
76.	Mammal	*Felis catus (Linnaeus, 1758) *Synonym-*Felis domesticus*	Cat	Least concern	
77.	Mammal	*Funambulus pennantii (Wroughton, 1905)*	Five Striped Palm Squirrel	Least concern	
78.	Mammal	*Hystrix indica (Kerr, 1792)*	Porcupine	Least concern	
79.	Mammal	*Lepus nigricollis (F. Cuvier, 1823)*	Hare	Least concern	
80.	Mammal	*Muntiacus muntjak (Zimmermann, 1780)*	Barking deer	Least concern	
81.	Mammal	*Oryctolagus cuniculus (Linnaeus, 1758)*	Hare	Least concern	
82.	Mammal	*Paraechinus micropus (Blyth, 1846)*	hedgehog	Least concern	
83.	Mammal	*Petaurista petaurista (Pallas, 1766)*	Flying squirrel	Least concern	
84.	Mammal	*Pseudois nayaur (Hodgson, 1833)*	Bharal	Least concern	
85.	Mammal	*Rattus rattus (Linnaeus, 1758)*	Rat	Least concern	
86.	Mammal	*Sus scrofa cristatus*	Indian Wild Boar	Least concern	
87.	Mammal	*Sus scrofa domestica*	Domesticated pig	Least concern	
88.	Mammal	*Semnopithecus entellus (Dufresne, 1797) *Synomym-*Presbytis entellus*	Hanuman Monkey	Least concern	I
89.	Mammal	*Ursus thibetanus (G. Cuvier, 1823) *Synonym-*Selenarctos thibetanus*	Himalayan black bear	Least concern	I
90.	Mammal	*Macaca mulatta (Zimmermann, 1780)*	Rhesus Macaque	Least concern	II
91.	Mammal	*Canis aureus (Linnaeus, 1758)*	Jackal	Least concern	III
92.	Mammal	*Herpestes edwardsii (É. Geoffroy Saint-Hilaire, 1818)*	Mongoose	Least concern	III
93.	Mammal	*Paradoxurus hermaphroditus (Pallas, 1777)*	Common Palm Civet, Toddy Cat	Least concern	III
94.	Mammal	*Bos taurus (Linnaeus, 1758) Synonym-Bos indicus*	Cattle		
95.	Mammal	*Equus caballus (Linnaeus, 1758)*	Horse		
96.	Mammal	*Homo sapiens (Linnaeus, 1758)*	Human		
97.	Mammal	*Canis lupus familiaris (Linnaeus, 1758) *Synonym-Canis familiaris	Dog		
98.	Mammal	*Hemitragus jemlahicus (H. Smith, 1826)*	Himalayan Thar	Near threatened	
99.	Mammal	*Hyaena hyaena (Linnaeus, 1758)*	Striped Hyena	Near threatened	
100.	Mammal	*Manis crassicaudata (Gray, 1827)*	Indian Pangolin	Near threatened	II
101.	Mammal	*Pteropus giganteus (Brünnich, 1782)*	Indian flying fox	Near threatened	II
102.	Mammal	*Equus onager khur (Lesson, 1827) Synonym-Equus hemionus khur (Lesson, 1827)*	Indian wild ass	Endangered	I
103.	Mammal	*Bos gaurus (H. Smith, 1827) *Synonym-*Bos frontalis*	Mithun	Vulnerable	
104.	Mammal	*Elephas maximus indicus (Cuvier, 1798)*	elephant	Vulnerable	I
105.	Mammal	*Melursus ursinus (Shaw, 1791)*	Sloth Bear	Vulnerable	I
106.	Mammal	*Moschus moschiferus (Linnaeus, 1758)*	Musk deer	Vulnerable	I
107.	Mammal	*Panthera pardus (Linnaeus, 1758)*	Leopard	Vulnerable	I
108.	Mammal	*Equus hemionus (Pallas, 1775)*	Indian wild ass	Vulnerable	II
109.	Mammal	*Semnopithecus johnii *Synonym-*Presbytis johni*	Black monkey	Vulnerable	II

## Result

**Figure 1:**
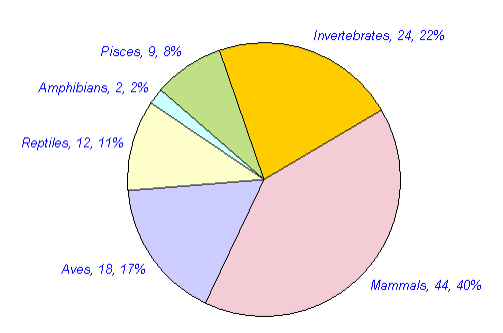
No. of animal species in different classes reported for medicinal purposes in India.

**Figure 2:**
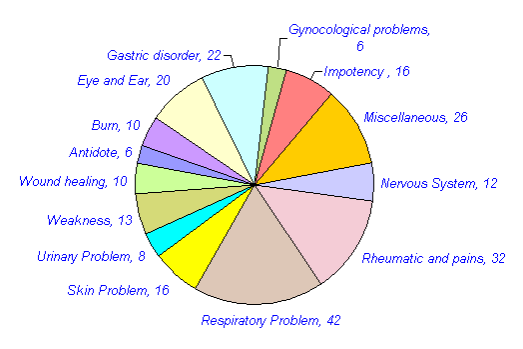
No. of animal species reported for medicinal uses in different disease categories in India.

**Figure 3:**
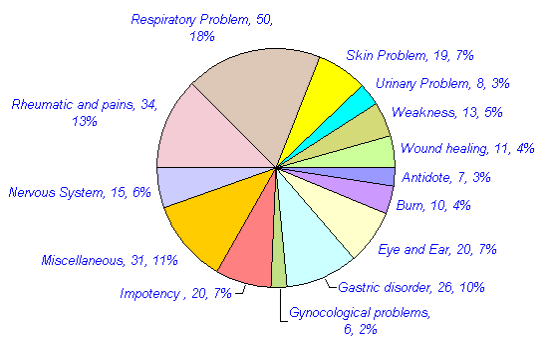
No. of medicinal uses reported in different disease categories in India.

**Figure 4:**
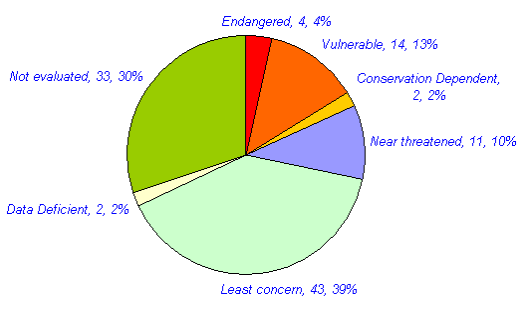
Conservation status of animal species reported for medicinal purposes in India according to IUCN Red List.

Approximately 109 animals are reported in traditional medicine in different parts of India. The mammals constitute the highest number of animals used for medicinal purposes. 44 (40%) mammals, 24 (22%) invertebrates, 18 (17%) birds, 12 (11%) reptiles, nine (8%) fishes and two (2%) amphibians have been reported for medicinal purposes (Table 3, figure 1). Approximately 270 medicinal uses of these animals are reported in different diseases in India. Many animals were used for the treatment of multiple ailments. Of these, the highest numbers of animal species (42, 38.5%) with 50 (18.5%) uses have been reported for the treatment of Respiratory system related problems. Rheumatic and other pains are treated with 32 species (29.4%) in 34 (12.9%) uses. Gastric problems are reported to be treated with 22 (20.2%) species in 26(9.9%) uses. Skin related Problems are treated with 16 species (14.7%) in 19 (7%) uses. 20 species (18.4%) are reported in 20 (7.6%) uses in Eye and Ear disease category. Impotency, aphrodisiac and birth control category is reported to be treated with 16 species (14.7%) in 20 (7.6%) uses. 26 (23.9%) animal species are reported in 31 (11.5%) uses in miscellaneous disease category (table 4, figure 2 and 3). Of the total 109 animal species reported, 76 (70%) are included in IUCN red data list (Table 5, figure 4). 36 (33%) animal species are listed in CITES appendix I, II, and III (Table 6).

**Table 3:** No. of animals species of different classes reported for medicinal purposes in India.

**Name of animal class**	**No. of species**	**% of Total animals**
**Mammals**	44	40%
**Aves**	18	17%
**Reptiles**	12	11%
**Amphibians**	2	2%
**Pisces**	9	8%
**Invertebrates**	24	22%
**Total**	109	

**Table 4:** No. of animal species and their medicinal uses reported in different disease categories in India.

**Disease Categories**	**No. of** **animal** **species** ** Uses**	**% of total** **109 animals** ** uses**	**No. of ** **medicinal** **applications** ** of animals**	**%**
**Antidote**	06	5.5%	07	2.7%
**Burn**	10	9.2%	10	3.8%
**Eye and Ear**	20	18.4%	20	7.6%
**Gastric disorder**	22	20.2%	26	9.9%
**Gynecological problems**	06	5.5%	06	2.3%
**Impotency, aphrodisiac, birth control**	16	14.7%	20	7.6%
**Miscellaneous**	26	23.9%	31	11.5%
**Nervous System**	12	11%	15	5.7%
**Rheumatic and other pains**	32	29.4%	34	12.9%
**Respiratory Problem**	42	38.5%	50	18.5%
**Skin related Problem**	16	14.7%	19	7%
**Urinary Problem**	8	7.3%	8	3%
**Weakness**	13	11.9%	13	5%
**Wound healing**	10	9.2%	11	4%
			270	

**Table 5:** Conservation status of animal species reported for medicinal purposes in India according to IUCN Red List or Red Data List.

**Conservation status**	**No. of animal species**	**% of total 109 animal** ** species reported**
**Endangered**	04	3.7%
**Vulnerable**	14	12.4%
**Conservation Dependent**	2	1.8%
**Near threatened**	11	10.1%
**Least concern**	43	39.4%
**Data Deficient**	2	1.8%
**Not evaluated**	33	
**Total**	109	70%

**Table 6:** Conservation status of animal species reported for medicinal purposes in India according to CITES.

**Appendix**	**CITES**	**% of the total animal** ** used**
**I**	11	10%
**II**	19	17.5%
**III**	6	5.5%
**Total**	36	33%

### Animal body part or product use as raw material

**Figure 5:**
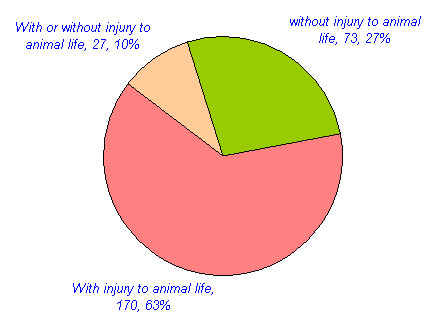
Raw material collected with or without injury to animal life for medicinal uses in India.

All animal body part or products use as raw materials are categorized in following three categories (Table 7, figure 5). (1) The flesh, fat, organs, bile blood, whole body and ash are those raw materials, which are always collected with injury to animal life. (2) But Excreta, urine, by-products (Honey, milk, mucous, wax, shellac, cocoon, musk, egg) are those raw materials, which are collected without injury to animal's life. (3) However some raw material like scale, antler, feather, teeth and bones can be collected with injury to animal life or some time these raw materials can be collected from natural dead animals.

**Table 7:** Raw material collected with or without injury to animal life for medicinal uses in India.

**Injury status**	**No. of medicinal uses**	**% of animal uses**
**With injury to animal life**	170	63%
**With or without injury to animal life**	27	10%
**without injury to animal life**	73	27%
**Total**	270	100%

**Figure 6:**
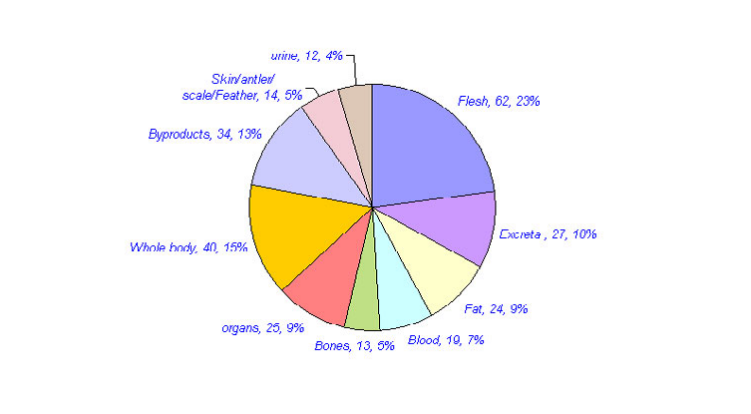
No. of animal part or products uses for medicinal purposes in different parts of India.

The raw materials are used in 170 medicinal preparations is always injured to animal life (flesh in 62 preparations, fat in 24 preparations, organs and bile in 25 preparations, blood in 19 preparations, whole body and ash in 40 preparations). The number of raw materials collected for medicinal preparation with injury to animal's life is very high (Table 8, figure 6). However in 73 medicinal uses, the raw materials are collected without injury to animal life (byproducts uses in 34 preparations, excreta uses in 27 preparations and urine uses in 12 preparations). Others 27 medicinal uses, the raw materials are collected with or without injury to animal life (scale, antler, feather, teeth are uses in 14 preparation and bones are uses in 13 preparations).

**Table 8:** Animal part or products uses for medicinal purposes in different parts of India.

**Medicinal uses without injury to animal**				**Medicinal uses ** **with/without injury to animal**		**Medicinal uses with injury to animals**				
**Disease**	**By-products** **(Honey, ** **milk,** ** mucous,** **wax, shellac,** **cocoon, musk, ** ** egg)**	**Excreta**	**Urine**	**scale/antler/** **Feather/teeth**	**Bones** **/carapace**	**Flesh** **/meat**	**Fat**	**Blood**	**Organs/** **bile**	**Whole ** **body/ash** **/powder**

**Antidote**							2		4	1
**Burn**					1		4	2	3	2
**Eye and Ear**	3		2	3	2	6			2	2
**Gastric disorder**	3	8	2	2	1	2			6	2
**Gynecological problems**	1	1			1	2				1
**Impotency**	1	4		1	1	12			1	
**Miscellaneous**	4	5	1	2	1	4	3	1	3	6
**Nervous System**		1	1			4		4	1	4
**Pain**	7	2	1			5	12	3	2	3
**Respiratory Problem**	7	4	2	3	2	16		5	3	9
**Skin related Problem**	3	1	1	3	2	2	2			4
**Urinary Problem**		2			1	3				2
**Weakness**	2		1			5		2		3
**Wound healing**	3		1		1	1	1	2		1

**Total-270**	**34**	**27**	**12**	**14**	**13**	**62**	**24**	**19**	**25**	**40**
**% of total-**	**13%**	**10%**	**4%**	**5%**	**5%**	**23%**	**8.9%**	**7%**	**9%**	**15%**

### Respiratory system Problems

The damp conditions in local homes, leading to high mold counts, as well as insufficient air circulation account for the prevalence of respiratory problems. Many houses in rural areas still have wooden stoves, with smoke causing constant irritation to the pulmonary system.

42 animal species with 50 uses is reported for the treatment of Respiratory related problems like asthma, cough, cold, tuberculosis in different part of India. Of the total 50 Respiratory uses, 32 uses are only for the treatment of asthma. In 16 uses, flesh of animal is reported as raw material for the treatment of respiratory problems. Because mostly ethnic communities eat flash of various animals to control asthma, so there can be a relation between animal flash and asthma.

### Gastric system Problems

Stomach disorders, liver problems, constipation, cholera, dysentery, etc are included in the gastric system problem category. 22 (20.2%) animal species are reported in 26 (9.9%) medicinal uses to treat gastric problems. Gastric problems treated include liver problems (2 uses); stomach problems (7 uses); constipation (2 uses); dysentery (3 uses); cholera (1 uses) and other gastric problems (2 uses).

The animal raw materials reported are urine, excreta, dung, feaces, Gall bladder bile, etc.

### Rheumatic and other pains

The housing conditions already described, as well as difficult working conditions, leads to a wide spectrum of pains. 34 uses (12.9%) of 32 animal species (29.4%) are fall into this illness category. Body pain, Sprain, Rheumatism, Muscle pain, Headache, Sprain, Bone fracture, Arthritis, Internal Pain, etc are included in this disease category. Animal raw materials are reported for the treatment of arthritis and rheumatic pain in the 23 uses. Mostly fat is uses as raw material in the pain related problems. Of the total 34 uses, fat is reported as raw material in 12 uses of this category. So there can be a relation between animal fat and pains.

### Skin related Problem

Skin infections, either fungal or bacterial, as well as sunspots, moles, pockmarks and acne can be observed frequently in India. Traditional healers are consequently consulted to treat these problems. 16 species (14.7%) are reported in 19 uses (7%) to treat skin problems. Fungal infections are particularly difficult to treat in the context of Western medicine, and the use of animal raw materials to alleviate such infections is thus of particular interest. Leprosy, Acne, leucoderma, Scabies, Spots, Itching, eczema, ringworm infections and to improve the fairness, etc are included in this disease category.

### Eye and Ear related Problem

20 (18.4%) animal species are reported for 20 (7.6%) uses to treat eye and ear related Problem. Eye-ache, Conjunctivitis, Night blindness, Cataract, Earache and pus in ear are included in this disease category. Legs of *Pavo cristatus (Linnaeus, 1758) *is used for ear infection is reported in many parts of India.

### Impotency, Aphrodisiac, Birth control

16 (14.7%) animal species are reported in 20 (7.6%) uses to treat Impotency and birth control related Problem in various part of India. This disease category included aphrodisiac, increase sexual desire and efficacy, birth control measure, male impotency and to attain early puberty. 19 uses are reported for increase sexual potency and two uses are for birth control measures. Four uses are for remove to male impotency. Sex organs mainly penis, excreta, flesh, etc are main animal raw materials uses in this category. Of the total 20 uses, 12 times flesh is reported as raw material in this disease category.

### Gynecological Problem

Gynecological problems are among the important medical problems treated by different *ethnic communities *of India. Infections of ovaries, uterus, and vagina as well as post partum infections were very common conditions for which women sought the help of healers. Six (5.5%) animal species are reported in six (2.3%) uses of Gynecological Problem in the various part of the India. Menstruation problem, Facilitates delivery, leucorrhoea, gonorrhea, etc are included in this disease category.

### Nervous System

The enormous role that traditional healer play in the area of treatment of psychosomatic and nervous system problems. 12 (11%) animal species are reported for 15 (5.7%) uses of nervous system disorders in the various part of the India. Epilepsy, paralysis migraine, nervous disorder, etc are main ailments that are included in this disease category. Of the total 15 uses, nine uses are reported for paralysis and four uses for epilepsy in this disease category.

### Weakness

13 (11.9%) animal species are reported in 13(5%) uses to treat weakness in the various part of the India. General weakness, anemia, malnutrition are main ailments, that are included in this disease category. In 13 uses, milk, flesh and blood are main raw materials reported in this category.

### Wound healing

Wound infections from accidents are very common in India, and are a major concern especially in rural areas. 10 (9.2%) animal species are reported for 11 (4%) uses of wound healing in the various parts of the India. small cuts, ulcers, wounds and mouth ulcers are included in this disease category.

### Urinary Problem

Eight (7.3%) animal species are reported for eight (3%) medicinal uses of urinary problems in the various parts of the India.

### Antidote

Six (5.5%) animal species are reported in seven (2.7%) uses to treat antidotes in the various parts of the India. Snake bite, spider bite, poisoning are included in this category. Bile duct, intestine, fat are reported as raw materials for antidote.

### Burn

10 (9.2%) animal species are reported for 10 (3.38%) medicinal uses of burn problems in the various parts of the India. Fat is mostly uses as raw material in burn wounds.

### Miscellaneous

26 (23.9%) animal species are reported for 31 (11.5%) uses of miscellaneous purposes in the various parts of the India. The rare disorders included are diabetes, Cancer, carbuncle, haematoma, eosinophilia, Enuresis (bed wetting), Internal tumours, Obesity, alcoholic drinks, Stammering, contracted limbs, hiccups, etc in this category.

## Discussion

It is widely accepted that plants, animals and their by-products used as a source of folk or traditional medicines indicate the presence of a biologically active constituent(s) in them. A significant portion of the currently available non-synthetic and/or semi-synthetic pharmaceuticals in clinical use is comprised of drugs derived from plants, animal, microbial, and mineral products [30-32]. Although today much is known about the phytochemistry and phytopharmacology of many traditional plant remedies, but real bio-scientific evaluations of remedies of animal origin are still quite rare in the literature [33]. However many animals have been methodically tested by pharmaceutical companies as sources of drugs to the modern medical science [34].

Approximately 109 animals and their 270 uses are reported in folk medicine in different part of India. The number of animals reported for medicinal purposes in different parts of India is enough to feel a need to discuss on the use of animals and their products, as medicines. In order to stress how important animals were, are and can be as sources of pharmacological substances and discussion on the use of the animals and their products, as medicines in conservation biology and sustainable use.

42 animal species with 50 uses is reported for the treatment of Respiratory problems like asthma, cough, cold, tuberculosis in different part of India. Of the total 50 Respiratory uses, 32 uses are only for the treatment of asthma. In 16 uses flesh of animal is reported as raw material for the use of respiratory problems. So there can be a relation between animal flash and asthma, because mostly ethnic communities reported flash of various animals is uses for asthma.

Kadrobova et al. (1996) reported that low selenium (Se) levels were observed in patients with asthma when compared to a group of patients without asthma. The researchers concluded that Se supplementation may be beneficial to patients with intrinsic asthma, who may be at risk of Se deficiency [35]. Selenium occurs in various chemical forms (selenite or selenate) in plants and animals. It is in an inorganic form such as selenomethionine or other selenium-containing amino acids [36]. The meat and fish group which include quantities of dry fish (*Tilapia nicotilus*), cray fish (*Procambaris clarkii*), snail (*Achatina fulica*) and albino rat was richest in selenium. Although snail and rat contained little or none [37].

In Brazil, Alves et al reported the medicinal uses of 283 animal species, 96% of which are wild caught and 27% of which are on one or more lists of endangered species [38]. Alves et al also demonstrate that at least 165 reptile species are used in traditional folk medicine around the world. Some species are used as sources of drugs for modern medical science. Of the reptiles recorded, 53% are included on lists of endangered species [39].

109 animal species are uses in India, of which 76 (70%) are included in IUCN red data list and 36 (33%) animal species are listed in CITES appendix I, II, and III and the Raw materials are used in 170 medicinal preparations is always injured to animal life. All these data is very high to affect biodiversity. Many protected animal species like peacock (*Pavo cristatus (Linnaeus, 1758)*, hard shelled turtle (*Kachuga tentoria (Gray, 1834)*), sambhar (*Cervus unicolor *(Kerr, 1792)), Spiny-tailed lizard (*Uromastyx hardwickii (Gray, 1827))*, and collared dove *(Streptopelia decaocto (Frivaldszky, 1838)) are *mentioned as important medicinal resources in India. The Kanjar community girls eat flesh of collared dove for attain puberty in early age in the surrounding areas of Ranthambhore national park [19]. Now collared dove facing a serious problem due to this activity in this area. It's suggested that this kind of neglected traditional knowledge should be included into the strategies of conservation and management of faunistic resources in the investigated areas.

## Conclusion

We have summarized and analyses the data collected by various authors in 15 published research works on zootherapeutic practices in different part of India. Some important points are outcome of this work.

1. Approximately 109 animals and their 270 medicinal uses are reported in traditional medicine in different parts of India.

2. Of the total 109 animal species reported, 76 (70%) are included in IUCN red data list. 36 (33%) animal species are listed in CITES appendix I, II, and III.

3. The mammals constitute the highest number of animals used for medicinal purposes. 44 (40%) mammals, 24 (22%) invertebrates, 18 (17%) birds, 12 (11%) reptiles, 9 (8%) fishes and two (2%) amphibians have been reported for medicinal purposes.

4. The highest numbers of animal species (42, 38.5%) with 50 (18.5%) uses have been reported for the treatment of Respiratory system related problems, like asthma, cough, cold, tuberculosis in different part of India. Of the total 50 Respiratory uses, 32 uses are only for the treatment of asthma. In 16 uses, flesh of animal is reported as raw material for the treatment of respiratory problems. Because mostly ethnic communities eat flash of various animals to control asthma, so there can be a relation between animal flash and asthma.

5. Rheumatic and other pains are reported to be treated with 32 species (29.4%) for 34 (12.9%) uses in different part of India.

6. Gastric problems are reported with 22(20.2%) for 26 (9.9%) uses in different part of India.

7. Skin related Problems are treated with 16 species (14.7%) for 19 (7%) uses in different part of India.

8. 20 species (18.4%) are used in 20 uses (7.6%) in eye and ear related diseases in different part of India.

9. Impotency, aphrodisiac and birth control is reported with 16 species (14.7%) for 20(19) (7.6%) uses in different part of India.

10. Raw materials are used in 170 medicinal preparations is always injured to animal life (flesh in 62 preparations, fat in 24 preparations, organs and bile in 25 preparations, blood in 19 preparations, whole body and ash in 40 preparations).

11. In 73 medicinal uses, the raw materials are collected without injury to animal life (byproducts uses in 34 preparations, excreta uses in 27 preparations and urine uses in 12 preparations). However in 27 medicinal uses, the raw materials are collected with or without injury to animal life (scale, antler, feather, teeth are uses in 14 preparation and bones are uses in 13 preparations).

12. Flesh is reported for maximum (62, 23%) uses as animal raw material in Indian ethnic communities.

## Supplementary Material

Additional file 1 Medicinal uses of animals and their products in different disease categories in India. All the medicinal uses of animals in India are classified in 14 major disease categories i.e. Antidote, Burn, Eye and Ear, Gastric disorder, Gynecological problems, Impotency, Nervous System, Pains, Respiratory Problem, Skin related Problem, Urinary Problem, Weakness and Wound healing. Each disease category table contains information in the following pattern: English name, scientific name, area or tribe reported, part or product or raw material name, mode of preparation and reference of the authors. Click here for file
