# Supplementary material for: Traditional zootherapeutic studies in India: a review
Source: J Ethnobiol Ethnomed. 2008 Jul 18;4:17. doi: 10.1186/1746-4269-4-17 (PMC2516508; doi:10.1186/1746-4269-4-17)
Supplement: Additional file 1 — Medicinal uses of animals and their products in different disease categories in India. All the medicinal uses of animals in India are classified in 14 major disease categories i.e. Antidote, Burn, Eye and Ear, Gastric disorder, Gynecological problems, Impotency, Nervous System, Pains, Respiratory Problem, Skin related Problem, Urinary Problem, Weakness and Wound healing. Each disease category table contains information in the following pattern: English name, scientific name, area or tribe reported, part or product or raw material name, mode of preparation and reference of the authors. [file 1746-4269-4-17-S1.pdf]

Additional file 1

Table1- animals and their products used in respiratory problems in India.

S. No.		English Name	*Scientific Name*	Tribe/ Inhabitant/ Region	Parts uses	Disease	Method of preparation and medicinal use	Related reported author in India
1.	Invertebrate	Honey bee	*Apis cerana indica (Fabricius 1798)*	Kanikaran tribes	Honey	Asthma, Cough and Cold	One gram of the shade-dried seeds powder mixture of *Piper nigrum* and *Piper longum,* leaves of *Adhatoda vasica* and *Ocium sanctum* and the roots of *Glycyrrhiza glabra, Alpinia galanga, Costus speceosus used with* 10^9 of natural honey.	Solavan A et al (2004)
2.	Invertebrate	Honey bee	*Apis cerana indica (Fabricius 1798)*	Tirunelveli district, Tamil Nadu	Honey	Cough, even Whooping cough, Asthma	It is given to old people and children every day by mixing it with water or milk. . It is also used as a vehicle for plant medicine.	Ranjit Singh et al (2004)
3.	Invertebrate	Honey bee	Apis florea (Fabricius, 1787)	Tirunelveli district, Tamil Nadu	Honey	Cough, even Whooping cough, Asthma	It is given to old people and children every day by mixing it with water or milk. . It is also used as a vehicle for plant medicine.	Ranjit Singh et al (2004)
4.	Invertebrate	Honey bee	Apis dorsata (Fabricius, 1793)	Tirunelveli district, Tamil Nadu	Honey	Cough, even Whooping cough, Asthma, Fever and Dyspepsia	It is given to old people and children every day by mixing it with water or milk. . It is also used as a vehicle for plant medicine.	Ranjit Singh et al (2004)
5.	Invertebrate	Cockroach	*Blatta orientalis Linnaeus, 1758*	Ao, Angami, Sema, Khiamniungan, Chakhesang, Lotha, Zeliang, Pochury, and Rengma Nagaland.	Whole body	Asthma and tuberculosis	Cockroaches are eaten raw as a remedy measure for relieving asthma and tuberculosis.	Jamir and Lal (2005)
6.	Invertebrate	Crab	*Cancer pagurus (Linnaeus, 1758)*	Saharia tribe	Whole body	Cough, asthma, tuberculosis	Ash of crab is used in lung diseases as cough, asthma, tuberculosis, etc.	Mahawar and Jaroli (2007)
7.	Invertebrate	Cochineal insect	*Dactylopius coccus (Costa, 1835)*	Tirunelveli district, Tamil Nadu	Whole body	Whooping cough	The insects are crushed, put into boiling water and then dried to prepare powder. The powder is used to treat whooping cough.	Ranjit Singh et al (2004)
8.	Invertebrate	Earthworm	*Pheretima posthuma (L. Vaillant, 1868)*	Kanikaran tribes	Mucous	Asthma	Five to six adult earthworms are subjected to ex-osmotic pressure for 2 hrs in 5 g of sugar to extract their	Solavan et al (2004)

							body fluid in the form of mucous.	
9.	Invertebrate	Pod Borer	Helicoverpa armigera (Hubner, 1805)	Chhattishgadh	Caterpillar	Asthma	Helicoverpa caterpillar feeding on Sphaeranthus leaves is used for the treatment of asthma.	Podia (1995)
10.	Invertebrate	Prawn	*Macrobrachium malcolmsonii (H. Milne-Edwards, 1844)*	Saharia tribe	Dried powder	Tuberculosis	Taken for cure of Tuberculosis.	Mahawar and Jaroli (2007)
11.	Invertebrate	Sandy shore Crab	Matuta planipes (Fabricius, 1798) Synonym- *Matuta victor*	Kachchh	Whole body	Asthma	Crab is boiled in water and then the inner watery part taken for asthma; boiled in oil and used externally for curing ear pains. Crab is eaten for the treatment of tuberculosis.	Gupta Leena et al (2003)
12.	Invertebrate	Green Leafhopper (GLH)	*Nephotettix nigropictus (Stal, 1870)*	Chhattishgadh	Whole body	Asthma	GLH with Kukronda (Blumea lacera) are used to reduce the intensity of asthma attack.	Oodhia (2003 c)
13.	Invertebrate	Apple Snail	Pila globosa (Swainson, 1822)	Ao, Angami, Sema, Khiamniungan, Lotha, Chakhesang, Sangtam, Pochury, Konyak and Rengma tribes of Nagaland.	Flesh	Asthma, Tuberculosis	Flesh is eaten after cooking as a relief measure in asthma, tuberculosis.	Jamir and Lal (2005)
14.	Invertebrate	Hermit Crab	*Uca pugnax*	Kachchh	Whole body	Asthma	Crab is boiled in water and then taken for asthma.	Gupta Leena et al (2003)
15.	Pisces	Eel	*Amphipnous cuchia (Hamilton, 1822).*	Ao Tribe of Nagaland	Blood	Asthma	Fresh blood is drunk.	Kakati et al (2006)
16.	Pisces	Eel Fish	*Monopterus albus (Zuiew, 1793)* Monopterus cuchia (Hamilton, 1822)	Ao, Angami, Sema, Khiamniungan, Lotha, Zeliang, Sangtam, Pochury, Konyak and Rengma tribes of Nagaland.	Blood	Asthma	Fresh blood is drunk to cure asthma.	Jamir and Lal (2005)
17.	Reptile	Hardshelled Turtle.	*Kachuga tentoria (Gray, 1834)*	Mogya, Bawaria, Meena and Saharia tribe of Rajasthan	Carapace	Cough, asthma, Tuberculosis	Ash of carapace is used in lung diseases as cough, asthma, tuberculosis, etc.	Mahawar and Jaroli (2006, 2007)
18.	Reptile	Indian Flap shell turtle	*Lissemys punctata (Lacépède, 1788)*	Kachchh	Carapace	Cough	Carapace is burnt and ash is used for healing of cough.	Gupta Leena et al (2003)
19.	Aves	Bank myna	*Acridotheres ginginianus (Latham, 1790)*	Kachchh	Flesh	Whooping cough	Roasted flesh is eaten to treat whooping cough.	Gupta Leena et al (2003)
20.	Aves	Pigeon	*Columba livia*	Ao Tribe of Nagaland	Flesh	Asthma	Soup is warmed and applied locally.	Kakati et al

			(Gmelin, 1789).					(2006)
21.	Aves	Crow	*Corvus splendens (Vieillot, 1817)*	Kachchh	Flesh	Whooping cough	Roasted or boiled flesh is eaten to treat whooping cough.	Gupta Leena et al (2003)
22.	Aves	Crow	*Corvus splendens (Vieillot, 1817)*	Chhattishgadh	Excreta	Cough	Few drops of juice of freshly collected excreta are sufficient to care chronic cough.	Oodhia (2003 b)
23.	Aves	Jungle fowl	*Gallus sonneratii (Temminck, 1813)*	Ao Tribe of Nagaland	Flesh	Asthma, Breathing problem	Properly cooked and eaten.	Kakati et al (2006)
24.	Aves	White-breasted Kingfisher	*Halcyon smyrnensis (Linnaeus, 1758)*	Bhil, Garasia, Kathodia of Rajasthan	Feather	Typhoid	Ash of feather and flesh is used in cure of typhoid.	Sharma (2002)
25.	Aves	White-breasted Kingfisher	*Halcyon smyrnensis (Linnaeus, 1758)*	Bhil, Garasia, Kathodia of Rajasthan	Flesh	Typhoid	Ash of feather and flesh is used in cure of typhoid.	Sharma (2002)
26.	Aves	House sparrow	*Passer domesticus (Linnaeus, 1758)*	Kachchh	Droppings	Asthma	Ash of excreta is used for treatment of asthma in children (three times a day with water for couple of days)	Gupta Leena et al (2003)
27.	Aves	Peacock	*Pavo cristatus (Linnaeus, 1758)*	Nandurbar district of Maharashtra	Feather	Cough	The ash of feather is used for cough.	Patil (2003)
28.	Aves	Red-wattled bird	*Vanellus indicus (Boddaert, 1783)*	Shoka tribe of Uttaranchal	Yolk egg	Typhoid	The yolk when applied on head is said to Lapwing cure typhoid. Often the local practitioners preserve the eggs within the cow dung for future use.	Negi and palyal (2007)
29.	Mammal	Bison	Bison bison (Linnaeus, 1758)	Chhattishgadh	Excreta	Asthma	The daily intake of excreta with honey in small dose is considered as beneficial to give strength to lungs and it is very useful in Chronic asthma.	Oodhia (2003 b)
30.	Mammal	Jackal	*Canis aureus (Linnaeus, 1758)*	Kachchh	Flesh	Asthma	Roasted flesh is eaten to cure asthma.	Gupta Leena et al (2003)
31.	Mammal	Jackal	*Canis aureus (Linnaeus, 1758) indicus*	Shoka tribe of Uttaranchal	Blood	Asthma	Blood is given to the patients suffering from asthma.	Negi and palyal (2007)
32.	Mammal	Goat	*Capra falconeri (Wagner, 1839)*	Shoka tribe of Uttaranchal	Flesh	Rhinitis	The meat is believed to stimulate digestion and cures rhinitis.	Negi and palyal (2007)
33.	Mammal	Goat	*Capra sibirica* (Pallas, 1776)	Ao, Angami, Sema, Khiamniungan, Chakhesang, Lotha, Zeliang, Sangtam, Pochury, Konyak and Rengma tribes of	Urine	Asthma, Tuberculosis	Drunk directly.	Kakati et al (2006), Jamir and Lal (2005)

				Nagaland.				
34.	Mammal	Bat	*Cynopterus sphinx (Vahl, 1797)*	Kachchh	Flesh	whooping cough	Raw flesh having blood is rubbed on the external injuries for healing and also eaten to treat whooping cough.	Gupta Leena et al (2003)
35.	Mammal	Hyena	*Hyaena hyaena (Linnaeus, 1758)*	Kachchh	Flesh	Asthma	Blood is applied on cloth and the dried cloth is fumigated to cure asthma.	Gupta Leena et al (2003)
36.	Mammal	Porcupine	*Hystrix indica (Kerr, 1792)*	Kachchh	Squills	Respiratory problems	Squills are fumigated for respiratory problems in children.	Gupta Leena et al (2003)
37.	Mammal	Porcupine	*Hystrix indica (Kerr, 1792)*	Shoka tribe of Uttaranchal	Stomach and intestinal parts are dried (with fecal matter),	Asthma	Stomach and intestinal parts are dried (along with the fecal matter), is given in the treatment of asthma.	Negi and palyal (2007)
38.	Mammal	Indian Hare	*Lepus nigricollis (F. Cuvier, 1823)*	Bhil, Garasia, Kathodia of Rajasthan	Ash	Cough	Ash of hare is given to cure cough.	Sharma (2002)
39.	Mammal	Indian Hare	*Lepus nigricollis (F. Cuvier, 1823)*	Bhil, Garasia, Kathodia of Rajasthan	Flesh	Typhoid	Flesh is eaten to treatment.	Sharma (2002)
40.	Mammal	Indian Hare	*Lepus nigricollis (F. Cuvier, 1823)*	Shoka tribe of Uttaranchal	Blood	Asthma	Blood of rabbit is used in asthma.	Negi and palyal (2007)
41.	Mammal	Monkey	*Macaca mulatta (Zimmermann, 1780)*	Shoka tribe of Uttaranchal	Flesh	Asthma,	The meat is believed to cure asthma.	Negi and palyal (2007)
42.	Mammal	Monkey	*Macaca (All species) Macaca mulatta (Zimmermann, 1780)*	Ao, Angami, Sema, Khiamniungan, Lotha, Zeliang, Sangtam, Pochury, Konyak and Rengma tribes of Nagaland.	Meat	Tuberculosis ,	Meat of all monkey species is eaten by Naga tribes. It is general belief that monkey meat cures tuberculosis.	Jamir and Lal (2005)
43.	Mammal	Bat	*Myotis lucifugus (LeConte, 1831)*	Ao, Angami, Sema, Khiamniungan, Chakhesang, Lotha, Zeliang, Pochury, Konyak and Rengma tribes of Nagaland., Kanikaran tribes	Flesh	Asthma,	Flesh is cooked and eaten for curing asthma.	Jamir and Lal (2005), Solavan et al (2004)
44.	Mammal	Sherni (Tigress)	Panthera tigris (Linnaeus, 1758)	Chhattishgadh	Milk	Asthma	This milk is also used in treatment of asthma.	Oodhia (2003 a)

45.	Mammal	Pale hedgehog	Paraechinus micropus (Blyth, 1846)	Kachchh	Skin	Respiratory problems and cold	Ash of skin is used for respiratory problems and cold.	Gupta Leena et al (2003)
46.	Mammal	Flying squirrel	*Petaurista petaurista (Pallas, 1766)*	Ao Tribe of Nagaland	Bile	Asthma	Boiled in water and drunk.	Kakati et al (2006)
47.	Mammal	Hanuman Monkey	Semnopithecus entellus (Defense, 1797) Synomyme- *Presbytis entellus*	Bhil, Garasia, Kathodia of Rajasthan	Urine	Severe Cough	A drop of urine mixed with 100 ml water is given in severe cough.	Sharma (2002)
48.	Mammal	Black monkey	Semnopithecus johnii Synonym- Presbytis johni	Kanikaran tribes	Blood	Asthma	The fresh blood is consumed as such by the tribal population. In some cases the meat of the monkey is cooked with the seeds of *Piper nigrum,* the stem of *Zingiber officinale,* root of the *Allium sativum.*	Solavan et al (2004)
49.	Mammal	Black monkey	Semnopithecus johnii Synonym- Presbytis johni	Kanikaran tribes	flesh	Asthma	The fresh blood is consumed as such by the tribal population. In some cases the meat of the monkey is cooked with the seeds of *Piper nigrum,* the stem of *Zingiber officinale,* root of the *Allium sativum.*	Solavan et al (2004)
50.	Mammal	Indian Flying Fox	*Pteropus giganteus (Brünnich, 1782)*	Bhil, Garasia, Kathodia of Rajasthan	Flesh	Asthma	Flesh is given to cure asthma.	Sharma (2002)

Table 2- Animals and their products used in human gastric problems in India,

**S.N.**		**English Name**	***Scientific Name***	**Tribe/ Inhabitant/ Region**	**Parts uses**	**Disease**	**Method of preparation and medicinal use**	**Related reported author in India**
1.	Invertebrate	Bed Bug	*Cimex lectularius (Linnaeus, 1758)*	Chhattishgadh	Whole body	Piles	External application on piles, inside the urethra (in case of urine retention).	Oodhia (1995)
2.	Invertebrate	Crab	*Cancer pagurus (Linnaeus, 1758)*	Ao, Angami, Sema, Khiamniungan, Chakhesang, Lotha, Zeliang, Sangtam, Yimchunger, Pochury, and	Whole body	Jaundice and other liver disorders	The whole body is crushed into a paste and boiled in water to prepare a drink which helps in curing Jaundice and other liver disorders.	Jamir and Lal (2005)

				Rengma tribes of Nagaland.				
3.	Invertebrate	Honey bee	*Apis cerana indica (Fabricius 1798)*	Tirunelveli district, Tamil Nadu	Wax	Fistula and piles	Applied externally.	Ranjit Singh et al (2004)
4.	Invertebrate	Honey bee	Apis dorsata (Fabricius, 1793)	Tirunelveli district, Tamil Nadu	Wax	Fistula and piles	Applied externally.	Ranjit Singh et al (2004)
5.	Invertebrate	Honey bee	Apis florea (Fabricius, 1787)	Tirunelveli district, Tamil Nadu	Wax	Fistula and piles	Applied externally.	Ranjit Singh et al (2004)
6.	Invertebrate	Apple Snail	Pila globosa (Swainson, 1822)	Ao, Angami, Sema, Khiamniungan, Chakhesang, Lotha, Sangtam, Pochury, Konyak and Rengma tribes of Nagaland.	Flesh	Stomach disorders.	Flesh is eaten after cooking as a relief measure in stomach disorders.	Jamir and Lal (2005)
7.	Reptile	Monitor	*Varanus bengalensis (Daudin, 1758)*	Ao Tribe of Nagaland	Skin	Piles	Cooked and eaten.	Kakati et al (2006)
8.	Aves	Pigeon	*Columba livia (Gmelin, 1789)*	Chhattishgadh	Excreta	Bleeding piles,	Use the excreta with the freshly collected flowers of Babool (Acacia nilotica).	Oodhia (2003 b)
9.	Aves	House sparrow	Passer domesticus (Linnaeus, 1758)	Mogya, Bawaria, Meena tribe of Rajasthan	Fecal	Constipation	Fecal matter is applied in the anus of baby to treat constipation.	Mahawar and Jaroli (2006)
10.	Aves	Barn or Screech Owl	*Tyto alba (Scopoli, 1769)*	Nandurbar district of Maharashtra	Wings	Stomach trouble	Babies with stomach trouble are exposed to smoke of wings.	Patil (2003)
11.	Mammal	Bison	Bison bison (Linnaeus, 1758)	Chhattishgadh	Excreta	Gastric troubles,	Half teaspoonful of excreta is recommended with a glass of hot milk before sleep till relief.	Oodhia (2003 b)
12.	Mammal	Cattle	Bos taurus (Linnaeus, 1758) *Synonym- Bos indicus*	Shoka tribe of Uttaranchal	Bile	Carminative	Gorocana or solidified cattle bile is cold in potency, use as carminative.	Negi and palyal (2007)
13.	Mammal	Camel	Camelus	Kachchh	Dung	Constipation	The dried dung is burnt and ash is	Gupta Leena et

			dromedarius (Linnaeus, 1758)				applied externally on stomach for treating constipation.	al (2003)
14.	Mammal	Jackal	*Canis aureus (Linnaeus, 1758)*	Maharashtra	Legs	Vomiting and dysentery	Smoke of legs is used for the cure of vomiting and dysentery.	Patil (2003)
15.	Mammal	Goat	*Capra sibirica* (Pallas, 1776)	Ao, Angami, Sema, Khiamniungan, Chakhesang, Lotha, Zeliang, Sangtam, Pochury, Konyak and Rengma tribes of Nagaland.	Urine	Stomach disorder	Drunk directly.	Kakati et al (2006), Jamir and Lal (2005)
16.	Mammal	Goat	*Capra sibirica* (Pallas, 1776)	Ao Tribe of Nagaland	Liver	Jaundice	Cooked and eaten.	Kakati et al (2006)
17.	Mammal	Goat	*Capra sibirica (Pallas, 1776)*	Ao, Angami, Sema, Khiamniungan, Chakhesang, Lotha, Zeliang, Sangtam, Pochury, Konyak and Rengma tribes of Nagaland.	Urine	Jaundice and other liver related problems	Urine is taken to cure stomachache. Also cures jaundice and other liver related problems.	Jamir and Lal (2005)
18.	Mammal	Indian ass	*Equus hemionus (Pallas, 1775)* ***Equus asinus*** (Linnaeus, 1758)	Mogya, Bawaria, Meena tribe of Rajasthan	Dung	Jaundice	Dung kept in water and after one day filtered water is given to cure jaundice.	Mahawar and Jaroli (2006)
19.	Mammal	porcupine	*Hystrix indica (Kerr, 1792)*	Bhil, Garasia, Kathodia of Rajasthan	Faeces	Gastric trouble	Faeces are given with water as pellet to infants.	Sharma (2002)
20.	Mammal	Porcupine	*Hystrix indica (Kerr, 1792)*	Ao Tribe of Nagaland	Bile	Dysentery	Soaked in rice, dried and taken.	Kakati et al (2006)
21.	Mammal	Porcupine	*Hystrix indica (Kerr, 1792)*	Ao, Angami, Sema, Khiamniungan, Lotha, Sangtam and Rengma tribes of Nagaland.	Intestin e	Cholera, dysentery and stomach disorders	A small portion of intestine is dried and then dipped overnight in cold water. This water is drunk to cure cholera, dysentery and stomach disorders.	Jamir and Lal (2005)
22.	Mammal	Indian Hare	*Lepus nigricollis (F. Cuvier, 1823)*	Nandurbar district of Maharashtra	Droppin g	Piles	The smoke of dropping is used for piles. Cattle suffering from foot and mouth disease are exposed to the smoke of legs.	Patil (2003)
23.	Mammal	Monkey	*Macaca (All species)*	Ao, Angami, Sema, Khiamniungan, Lotha,	Meat	Stomach disorder	Meat of all monkey species is eaten by Naga tribes. It is general belief	Jamir and Lal (2005)

			Macaca mulatta (Zimmermann , 1780)	Zeliang, Sangtam, Pochury, Konyak and Rengma tribes of Nagaland.			that monkey meat cures stomach disorder.	
24.	Mammal	Bharal	*Pseudois nayaur (Hodgson, 1833)*	Shoka tribe of Uttaranchal	Antlers	Stomach pain	Antlers are rubbed to produce a paste, which is either drunk or applied on the stomach to get relieved of stomach pain or fever.	Negi and palyal (2007)
25.	Mammal	Flying Fox	*Pteropus giganteus (Brünnich, 1782)*	Nandurbar district of Maharashtra	Bone	Stomach problems	The bone of bat is heated and applied to belly for stomach problems in infants. Droppings are fed to persons addicted to liquor.	Patil (2003)
26.	Mammal	Rat	*Rattus rattus (Linnaeus, 1758)*	Chhattishgadh	Excreta (dried)	Bleeding pile,	The rat excreta (dried) is recommended with the fresh juice of Kukronda (Blumea lacera) leaves externally. Patients are advised to put this mixture inside the anus for quick and permanent relief.	Oodhia (2003 b)

Table 3- Animals and their products used in human diverse kind of pains in India.

S. No.		English Name	*Scientific Name*	Tribe/ Inhabitant/ Region	Parts uses	Disease	Method of preparation and medicinal use	Related reported author in India
1.	Invertebrate	Honey bee	*Apis cerana indica (Fabricius 1798)*	Tirunelveli district, Tamil Nadu	Wax	Rheumatic pain.	Applied externally.	Ranjit Singh et al (2004)
2.	Invertebrate	Honey bee	Apis dorsata (Fabricius, 1793)	Tirunelveli district, Tamil Nadu	Wax	Rheumatic pain.	Applied externally.	Ranjit Singh et al (2004)
3.	Invertebrate	Honey bee	Apis florea (Fabricius, 1787)	Tirunelveli district, Tamil Nadu	Wax	Rheumatic pain.	Applied externally.	Ranjit Singh et al (2004)
4.	Invertebrate	Lac insect	Kerria lacca (Kerr, 1782) *Synonym- Laccifer lacca*	Tirunelveli district, Tamil Nadu	shellac	Rheumatism	Powdered shellac is mixed with honey and is used as remedy for caries. "*Lakshadi Thaila*", used to bring down chronic fevers and to	Ranjit Singh et al (2004)

							cure rheumatism has shellac as its main ingredient.	
5.	Invertebrate	Scorpion	*Heterometrus swammerdami (Simon, 1872) Synonym - Palamnaeus swammerdami*	Ao, Angami, Sema, Khiamniungan, Chakhesang, Lotha, Zeliang, Sangtam, Konyak tribes of Nagaland.	whole body	Rheumatic joints pain	The whole body is boiled in mustard oil and the extract so prepared is used for massaging to relieve rheumatic joints pain.	Jamir and Lal (2005)
6.	*Pisces*	*Channa*	Channa punctata (Bloch, 1793) *Synonym- Channa punctatus Linn.*	Dibrugarh District, Assam	Edible portion of the fish	Body pain	Curry prepared from fishes.	Kalita (2005)
7.	*Pisces*	Hammer head shark	Eusphyra blochii (Cuvier, 1816) Synonym- Zygaena blochii	Kachchh	Fat	Joints pain	Fat is applied externally for treating joints pain.	Gupta Leena et al (2003)
8.	Amphibia	Frog	Fejervarya limnocharis synonym- *Lymnonecties limnorcharis*	Ao, Angami, Sema, Khiamniungan, Chakhesang, Lotha, Zeliang, Sangtam, Pochury, Konyak and Rengma tribes of Nagaland.	Fat	Rheumatic-joints pain	Fat is used for massaging rheumatic-joints to relieve pain.	Jamir and Lal (2005)
9.	Reptile	Cobra	*Naja naja (Linnaeus, 1758)*	Ao, Sema, Khiamniungan, Chakhesang, Lotha, Zeliang, Sangtam, Pochury, Konyak tribes of Nagaland.	Fat	Body ache, rheumatic and burn wounds pain	Fat is applied to relieve body ache, rheumatic and burn wounds pain and also for rapid healing of fractured bones	Jamir and Lal (2005)
10.	Reptile	Python	Python reticulatus (Schneider, 1801)	Ao, Angami, Sema, Khiamniungan, Chakhesang, Zeliang, Sangtam, Pochury, Konyak and Rengma tribes of Nagaland.	Fat	Body ache, rheumatic, and burn wounds pain	Fat is applied to relieve body ache, rheumatic, and burn wounds pain.	Jamir and Lal (2005)
11.	Reptile	Spiny tailed lizard	*Uromastyx hardwickii (Gray, 1827)*	Kachchh	Whole body	Joint pain, Rheumatism	The whole animal is boiled in oil and the oil applied externally.	Gupta Leena et al (2003)
12.	Reptile	Monitor	*Varanus bengalensis (Daudin, 1758)*	Ao Tribe of Nagaland Nandurbar district of Maharashtra	Fat	Rheumatism, Body pain, Piles,	The fat (oil) of this lizard is used for pain.	Kakati et al (2006), Patil (2003)

13.	Reptile	Monitor	*Varanus salvator (Laurenti, 1768)*	all the Tamilnadu tribes except Toda	flesh	Arthritis,	One kg of the meat along with the skeleton of the monitor is mixed together with 3 liter of coconut milk and boiled for 48 hrs until the meat is completely dissolved. After removing the bones from the medium 50g of ganja leaf *(Cannabis sativa)* is added to the preparation and boiled until it solidifies.	Solavan et al (2004)
14.	Aves	Crow-phesant	*Centropus sinensis (Stephens, 1815)*	Ao, Angami, Sema, Khiamniungan, Chakhesang, Lotha, Zeliang, Sangtam, Pochury, Konyak and Rengma tribes of Nagaland.	Flesh	Bodyache and Rheumatic pain	Flesh is eaten as food to cure bodyache and rheumatic pain.	Jamir and Lal (2005)
15.	Aves	Hen	*Gallus gallus domesticus*	Kachchh	Blood	Body pain<br><br>Arthritis	Blood is externally massaged on the aching parts of body.<br><br>Blood is applied externally for healing arthritis	Gupta Leena et al (2003)
16.	Mammal	Bison	Bison bison (Linnaeus, 1758)	Chhattishgadh	Excreta	Internal injury with severe pain	Excreta are given to patient with water.	Oodhia (2003 b)
17.	Mammal	Mithun	*Bos gaurus (H. Smith, 1827)* Synonym- *Bos frontalis*	Ao, Angami, Sema, Khiamniungan, Chakhesang, Lotha, Zeliang, Sangtam, Yimchunger, Pochury, and Rengma tribes of Nagaland.	Male organ (Penis)	Breast pain of lactating mother	Male organ is cooked and eaten to relieve severe pain in breast of lactating mother.	Jamir and Lal (2005), Kakati et al (2006)
18.	Mammal	Cow	Bos taurus (Linnaeus, 1758) *Synonym- Bos indicus*	Mogya, Bawaria, Meena tribe of Rajasthan	Dung + Milk	Muscle pain	Muscle pain can relieve by smear of dung and milk mixture.	Mahawar and Jaroli (2006)
19.	Mammal	Buffalo	*Bubalus bubalis (B. arnee) (Linnaeus, 1758)*	Ao Tribe of Nagaland	Fat	Body pain, Sprain, Rheumatism	Applied externally as embrocation/ massager.	Kakati et al (2006)
20.	Mammal	Camel	*Camelus dromedarius (Linnaeus, 1758)*	Saharia tribe	Milk	Muscular pain	Used as massage cream in muscular pain.	Mahawar and Jaroli (2007)

21.	Mammal	Jackal	*Canis aureus (Linnaeus, 1758)*	Shoka tribe of Uttaranchal	Flesh	Arthritis	The meat is believed to cure arthritis.	Negi and palyal (2007)
22.	Mammal	Dog	Canis lupus familiaris (Linnaeus, 1758) Synonym- Canis familiaris	Ao, Angami, Sema, Khiamniungan, Chakhesang, Lotha, Zeliang, Sangtam, Pochury, Konyak and Rengma tribes of Nagaland.	Urine	Headache	Urine cures is applied on fore head to relieve headache.	Jamir and Lal (2005)
23.	Mammal	Goat	*Capra sibirica* (Pallas, 1776)	Ao Tribe of Nagaland	Legs	Sprain, Bone fracture	Lower portion of legs cooked.	Kakati et al (2006)
24.	Mammal	Indian wild ass	*Equus hemionus khur* (Lesson, 1827)	Kachchh	Blood	Arthritis	Blood is massaged externally for healing arthritis	Gupta Leena et al (2003)
25.	Mammal	Cat	Felis catus (Linnaeus, 1758) Synonym- *Felis domesticus*	Shoka tribe of Uttaranchal	Whole body	Arthritis	The whole animal is stripped off and boiled and the resultant juice is said to cure arthritis.	Negi and palyal (2007)
26.	Mammal	Striped Hyena	*Hyaena hyaena (Linnaeus, 1758)*	Bhil, Garasia, Kathodia of Rajasthan Kachchh	Fat	Arthritis	Fat is applied externally in massage in arthritis.	Sharma (2002), Gupta Leena et al (2003)
27.	Mammal	Porcupine	*Hystrix indica (Kerr, 1792)*	Ao, Angami, Sema, Khiamniungan, Lotha, Sangtam and Rengma tribes of Nagaland.	Fat	Bodyache, rheumatic pain,	Fat is applied to relieve bodyache, rheumatic pain.	
28.	Mammal	Monkey	*Macaca mulatta (Zimmermann, 1780)*	Shoka tribe of Uttaranchal	Flesh	Rheumatism,	The meat is believed to cure rheumatism.	Negi and palyal (2007)
29.	Mammal	Panther	*Panthera pardus (Linnaeus, 1758)*	Bhil, Garasia, Kathodia of Rajasthan and Shoka tribe of Uttaranchal	Fat	Arthritis body pain	Fat is used in massage in arthritis. The fat is used as massaging oil in the treatment of body pain.	Sharma (2002), Negi and palyal (2007)
30.	Mammal	Tiger	*Panthera tigris (Linnaeus, 1758)*	Ao, Angami, Sema, Khiamniungan, Chakhesang, Lotha, Zeliang, Sangtam, Pochury, Konyak and Rengma tribes of Nagaland., Bhil, Garasia, Kathodia of	Fat	Arthritis bodyache and rheumatic pain.	Fat is applied for relieving bodyache and rheumatic pain.	Jamir and Lal (2005), S. K. Sharma

				Rajasthan				
31.	Mammal	Sherni (Tigress)	Panthera tigris (Linnaeus, 1758)	Chhattishgadh	milk	Pain breasts	For external application, they massage it on breasts.	Oodhia (2003 a)
32.	Mammal	Pig	Sus scrofa *domestica*	Mogya, Bawaria, Meena tribe of Rajasthan Ao Tribe of Nagaland	Fat	Body pain	Fat of pig is use as massage cream in muscular pain.	Mahawar and Jaroli (2006), Kakati et al (2006)
33.	Mammal	Indian Wild boar	*Sus scrofa cristatus*	Bhil, Garasia, Kathodia of Rajasthan	Blood	Internal pain	Blood or dry blood powder is mixed with 'mahuri' (local liqure prepare by flower of Madhuca indica) is given to cure internal pain.	Sharma (2002)
34.	Mammal	Indian Wild Boar	Sus scrofa *cristatus*	Nandurbar district of Maharashtra	oil	Rheumatic pains	The oil is used for joint (rheumatic) pains.	Patil (2003)

Table 4- Animals and their products used in nervous system related problems of India.

S. No.		English Name	*Scientific Name*	Tribe/ Inhabitant/ Region	Parts uses	Disease	Method of preparation and medicinal use	Related reported author in India
1.	Invertebrate	Bed Bug	*Cimex lectularius (Linnaeus, 1758)*	Chhattishgadh	Whole body	Epilepsy		Oodhia (1995)
2.	Invertebrate	Velvet ant	*Dasymutilla occidentalis (Linnaeus)*	Tirunelveli district, Tamil Nadu	Whole body	Paralysis	The powder of this ant is mixed with honey and given to patients suffering from paralysis.	Ranjit Singh et al (2004)
3.	Invertebrate	Pod Borer	*Helicoverpa armigera (Hubner, 1805)*	Chhattishgadh	Caterpillar	Nervous breakdown	Villagers use powder (after drying and crushing) of caterpillars as a tonic.	Oodhia (1995)
4.	Invertebrate	Red Velvet Mite	*Trombidium grandissimum (Koch, 1867)*	Chhattishgadh	Whole body	Paralysis		Oodhia (1995)
5.	Aves	pigeon	*Columba livia (Gmelin, 1789)*	most of the Tamilnadu tribes	Blood	Epilepsy	Fresh blood of the pigeon is applied on the skin from head to foot thrice a week. The blood should remain on the skin for 6 hrs.	Solavan et al (2004)
6.	Aves	Pigeon	*Columba livia (Gmelin, 1789)*	Bhil, Garasia, Kathodia, Saharia of Rajasthan and Shoka tribe of Uttaranchal	Flesh	Paralysis	Flesh is eaten to cure Paralysis.	Sharma (2002), Negi and palyal (2007), Mahawar and Jaroli (2007)

7.	Aves	Pigeon	*Columba livia (Gmelin, 1789)*	Mogya, Bawaria, Meena, Saharia tribe of Rajasthan most of the Tamilnadu tribes Kachchh	blood fresh	Paralysis	The fresh blood is massaged externally to treat paralysis. Fresh blood of the pigeon is applied on the skin from head to foot thrice a week. The blood should remain on the skin for 6 hrs.	Mahawar and Jaroli (2006) (2007), Solavan et al (2004), Gupta Leena et al (2003)
8.	Aves	Pigeon	*Columba livia (Gmelin, 1789)*	Chhattishgadh	Excreta	Migraine	Excreta of pigeon in combination with mustard seed for external application.	Oodhia (2003 b)
9.	Aves	Crow	Corvus splendens (Vieillot, 1817)	Ao Tribe of Nagaland	Flesh	Paralysis	Cooked and eaten.	Kakati et al (2006)
10.	Mammal	Jackal	*Canis aureus (Linnaeus, 1758)*	Shoka tribe of Uttaranchal	Flesh	Paralysis	The meat is believed to cure paralysis.	Negi and palyal (2007)
11.	Mammal	Goat	*Capra sibirica* (Pallas, 1776)	Ao, Angami, Sema, Khiamniungan, Chakhesang, Lotha, Zeliang, Sangtam, Pochury, Konyak and Rengma tribes of Nagaland.	Urine	Paralysis disorder	Drunk directly.	Kakati et al (2006), Jamir and Lal (2005)
12.	Mammal	Five-Striped Palm Squirrel	*Funambulus pennantii (Wroughton, 1905)*	Bhil, Garasia, Kathodia of Rajasthan	Flesh	Epilepsy	Flesh is eaten to cure epilepsy.	Sharma (2002)
13.	Mammal	Striped Hyena	*Hyaena hyaena (Linnaeus, 1758)*	Nandurbar district of Maharashtra	Brain	Epilepsy	Brain mixed with wheat flour is used to cure epilepsy.	Patil (2003)
14.	Mammal	Rhesus Macaque	*Macaca mulatta (Zimmermann, 1780)*	Bhil, Garasia, Kathodia of Rajasthan	Blood	Paralysis	Blood is given to cure paralysis.	Sharma (2002)
15.	Mammal	Black ibis	*Pseudibis papillosa (Temminck, 1824)*	Kachchh	Blood	Nervous disorder, paralysis	Blood is massaged for curing nervous disorder, paralysis and inherent body heat.	Gupta Leena et al (2003)

Table 5- Animals and their products used in General weakness in India

S.N.		English Name	*Scientific Name*	Tribe/ Inhabitant/ Region	Parts uses	Disease	Method of preparation and medicinal use	Related reported author in India
1.	Invertebrate	Silkworm	*Bombyx mori (Linnaeus)*	Tirunelveli district, Tamil Nadu	Larvae	Nutrient food	After boiling, the killed larvae are taken as nutrient food by some local people.	Ranjit Singh et al (2004)
2.	Invertebrate	Ant	*Dorylus labiatus (Shuckard, 1840)*	Tirunelveli district, Tamil Nadu	Whole body	Good health	These ants are collected, put into honey and after a fortnight the honey is taken to strengthen nervous system and to restore good health.	Ranjit Singh et al (2004)
3.	Invertebrate	Pod Borer	*Helicoverpa armigera (Hubner, 1805)*	Chhattishgadh	Caterpillar	Fever, general weakness,	Villagers use powder (after drying and crushing) of caterpillars as a tonic. They generally apply the fresh extracts of caterpillars on injured parts of the body to stop bleeding as a first aid measure.	Oodhia (1995)
4.	Pisces	Eel	*Amphipnous cuchia (Hamilton, 1822).*	Ao Tribe of Nagaland	Blood	General weakness	Fresh blood is drunk.	Kakati et al (2006)
5.	Pisces	Eel Fish	*Monopterus albus (Zuiew, 1793)* Monopterus cuchia (Hamilton, 1822)	Ao, Angami, Sema, Khiamniungan, Lotha, Zeliang, Sangtam, Pochury, Konyak and Rengma tribes of Nagaland.	Blood	General weakness, anemia	Fresh blood is drunk to cure general weakness and to relieve anemia.	Jamir and Lal (2005)
6.	Aves	Pigeon	*Columba livia (Gmelin, 1789).*	Ao, Angami, Sema, Khiamniungan, Lotha, Zeliang, Pochury tribes of Nagaland.	Flesh	General weakness	Flesh of Young birds is cooked and eaten for early recovery.	Kakati et al (2006), Jamir and Lal (2005)
7.	Aves	Crow	*Corvus splendens (Vieillot, 1817)*	Palliyan, Irular and Sholaga tribes	Flesh	Anemia	The flesh is cooked along with the seeds of the *Piper nigrum* and *Piper longum*; stem of ginger *Zingiber officinale;* root of *Allium sativum;* ghee of cow and the natural honey to make a 'legheum.'	Solavan et al (2004)

8.	Aves	house sparrow	*Passer domesticus (Linnaeus, 1758)*	Kanikaran, Irular and Kurimba tribes	Flesh	Anemia	The flesh is cooked along with the seeds of the *Piper nigrum* and *Piper longum*; stem of ginger *Zingiber officinale;* root of *Allium sativum;* ghee of cow and the natural honey to make a 'legheum'.	Solavan et al (2004)
9.	Mammal	Cow	Bos taurus (Linnaeus, 1758) *Synonym- Bos indicus*	Mogya, Bawaria, Meena tribe of Rajasthan	Urine	Weakness	Weakness due to fever is cure by drinking urine.	Mahawar and Jaroli (2006)
10.	Mammal	Goat	*Capra sibirica* (Pallas, 1776)	Ao Tribe of Nagaland	Milk	General Weakness	Drunk directly.	Kakati et al (2006)
11.	Mammal	Goat	*Capra sibirica (Pallas, 1776)*	Ao, Angami, Sema, Khiamniungan, Chakhesang, Lotha, Zeliang, Sangtam, Pochury, Konyak and Rengma tribes of Nagaland.	Milk	Weakness	Milk is drunk to avoid weakness.	Jamir and Lal (2005)
12.	Mammal	Monkey	*Macaca (All species) Macaca mulatta (Zimmermann, 1780)*	Ao, Angami, Sema, Khiamniungan, Lotha, Zeliang, Sangtam, Pochury, Konyak and Rengma tribes of Nagaland.	Meat	General weakness	Meat of all monkey species is eaten by Naga tribes. It is general belief that monkey meat cures general weakness.	Jamir and Lal (2005)
13.	Mammal	Common Palm Civet, Toddy Cat	*Paradoxurus hermaphroditus (Pallas, 1777)*	Nandurbar district of Maharashtra	Meat	Weakness	Meat is used for feeding weak cattle.	Patil (2003)

Table 6- Animals and their products used in urinary problems in India.

**S. N.**		**Name English**	***Scientific Name***	**Tribe/ Inhabitant/ Region**	**Parts uses**	**Disease**	**Method of preparation and medicinal use**	**Related reported author in India**
**1.**	Invertebrate	Bed Bug	*Cimex lectularius (Linnaeus, 1758)*	Chhattishgadh	Whole body	Urinary disorders	Application inside the urethra (in case of urine retention).	Oodhia (1995)
2.	Invertebrate	Red	*Trombidium*	Chhattishgadh	Whole	Urinogenital		Oodhia (1995)

		Velvet Mite	*grandissimum (Koch, 1867)*		body	disorders		
3.	Pisces	Labeo	*Labeo rohita (Hamilton, 1822)*	Saharia tribe	Cervical vertebrae	Urine Problem	A fish cervical vertebra is rubbed with water and this essence water is taken in urine blockage problem.	Mahawar and Jaroli (2007)
4.	Reptile	Snakes	Gloydius himalayanus (Günther, 1864) Synonym- *Ancistrodon himalayans*	Shoka tribe of Uttaranchal	Flesh	Elimination of urine	The meat is believed to facilitate the elimination of urine, stool and flatus.	Negi and palyal (2007)
5.	Reptile	Snakes	*Ptyas mucosus (Linnaeus, 1758)*	Shoka tribe of Uttaranchal	Flesh	Elimination of urine	The meat is believed to facilitate the elimination of urine, stool and flatus.	Negi and palyal (2007)
6.	Reptile	Snakes	Daboia russelii (Shaw & Nodder, 1797) Synonym- *Vipera russelli*	Shoka tribe of Uttaranchal	Flesh	Elimination of urine	The meat is believed to facilitate the elimination of urine, stool and flatus.	Negi and palyal (2007)
7.	Aves	Pigeon	*Columba livia (Gmelin, 1789)*	Chhattishgadh	Excreta	Kidney stones	The use of excreta with sugar is very effective in order to drain away the kidney stones.	Oodhia (2003 b)
8.	mammal	Cow	Bos taurus (Linnaeus, 1758) *Synonym- Bos indicus*	Kachchh	Dung	Urticaria	The dried dung is burnt and ash is applied externally to treat urticaria.	Gupta Leena et al (2003)

Table 7- Animals and their products used in impotency, aphrodisiac and birth control in India.

**S.N.**		**Name English**	***Scientific Name***	**Tribe/ Inhabitant/ Region**	**Parts uses**	**Disease**	**Method of preparation and medicinal use**	**Related reported author in India**
1.	Aves	Chir pheasant	*Catreus wallichii (Hardwicke, 1827)*	Shoka tribe of Uttaranchal	Flesh	Strength and semen.	The meat is believed to be a promoter of strength and semen.	Negi and palyal (2007)
2.	Aves	Indian Peafowl	*Pavo cristatus (Linnaeus, 1758)*	Saharia tribe	Feather	Infertility	Rounded spots of feather mix with Jaggery.	Mahawar and Jaroli (2007)
3.	Aves	Peacock	*Pavo cristatus*	Ao, Angami, Sema,	Flesh	Aphrodisiac	Flesh is cooked and eaten as an	Jamir and Lal

			(Linnaeus, 1758)	Khiamniungan, Lotha, Zeliang, Konyak tribes of Nagaland			aphrodisiac.	(2005)
4.	Aves	Collared dove	Streptopelia decaocto (Frivaldszky, 1838)	Mogya, Bawaria, Meena tribe of Rajasthan	Flesh	To attain early puberty	To attain early puberty girls eat flesh of collared dove.	Mahawar and Jaroli (2006)
5.	Aves	Owl	Strix aluco nivicolum (Blyth, 1845)	Shoka tribe of Uttaranchal	Flesh	Strength and virility	The meat is believed to be a promoter of strength and virility.	Negi and palyal (2007)
6.	Mammal	Cow	Bos taurus (Linnaeus, 1758) *Synonym- Bos indicus*	Chhattishgadh	milk	Good sexual health	Teaspoonful of Musli root powder with a glass of cow milk daily in order to maintain the sexual health.	Oodhia (2003 a)
7.	Mammal	Dog	Canis lupus familiaris (Linnaeus, 1758) Synonym- Canis familiaris	Ao, Angami, Sema, Khiamniungan, Chakhesang, Lotha, Zeliang, Sangtam, Pochury, Konyak and Rengma tribes of Nagaland.	Flesh	Male Impotency, pre and post-partum health	Flesh soup improves pre and post-partum health. Flesh soup cures male Impotency.	Jamir and Lal (2005)
8.	Mammal	Elephant	*Elephas maximus indicus (Cuvier, 1798)*	Chhattishgadh	Excreta	Birth control measure	Female patient to put a piece of elephant excreta inside the vagina in order to prevent pregnancy.	Oodhia (2003 b)
9.	Mammal	Donkey	*Equus hemionus (Pallas, 1775)*	Chhattishgadh	Excreta	Sexual desire and efficacy	The use of the excreta to increase the sexual desire and efficacy.	Oodhia (2003 b)
10.	Mammal	Horse	*Equus caballus (Linnaeus, 1758)*	Shoka tribe of Uttaranchal	Flesh	Corpulence, strength	Meat is believed to be a promoter of corpulence, strength.	Negi and palyal (2007)
11.	Mammal	Himalayan Thar	*Hemitragus jemlahicus (H. Smith, 1826)*	Shoka tribe of Uttaranchal	Flesh	Strength and virility	The meat is believed to be a promoter of strength and virility (usually that of the tail).	Negi and palyal (2007)
12.	Mammal	Sloth Bear	*Melursus ursinus (Shaw, 1791)*	Nandurbar district of Maharashtra	Sex organs	Aphrodisiac	The sex organs are used as aphrodisiac. The hair is used for prevention of fear in infants. Tail is hanged on front door to protect the house from evil.	Patil (2003)
13.	Mammal	Barking deer	*Muntiacus muntjak (Zimmermann, 1780)*	Shoka tribe of Uttaranchal	Flesh	Strength and virility	The meat is believed to be a promoter of strength and virility and is a good cardiac tonic.	Negi and palyal (2007)
14.	Mammal	Bat	*Myotis lucifugus (LeConte, 1831)*	Ao, Angami, Sema, Khiamniungan,	Flesh	Male impotency	Flesh is cooked and eaten for curing male impotency.	Jamir and Lal (2005)

				Chakhesang, Lotha, Zeliang, Pochury, Konyak and Rengma tribes of Nagaland.				
15.	Mammal	Leopard	*Panthera pardus (Linnaeus, 1758)*	Shoka tribe of Uttaranchal	Bones	Aphrodisiac	The bones are Supposed to be an aphrodisiac.	Negi and palyal (2007)
16.	Mammal	Leopard	*Panthera pardus (Linnaeus, 1758)*	Shoka tribe of Uttaranchal	Flesh	Strength and virility	The meat is believed to be a promoter of strength and virility.	Negi and palyal (2007)
17.	Mammal	Rat	*Rattus rattus (Linnaeus, 1758)*	Shoka tribe of Uttaranchal	Flesh	Promoter of semen	The meat is believed to be a promoter of semen.	Negi and palyal (2007)
18.	Mammal	Rat	*Rattus rattus (Linnaeus, 1758)*	Chhattishgadh	Excreta	Birth control measure,	The traditional healers advise the women to put the rat excreta mixed with honey and the fresh leaf juice of Mucuna leaves inside the vagina.	Oodhia (2003 b)
19.	Mammal	Rat	*Rattus rattus (Linnaeus, 1758)*	Chhattishgadh	Excreta	Increase your sexual desire and efficacy	Dried excreta (not sun dried; dried in shady places) is mixed with any one of these liquids (water, milk Mahua liquor, honey , etc.) and applied on male genital just before intercourse. The liquids are	Oodhia (2003 b)
20.	Mammal	Wild Boar	*Sus scrofa cristatus*	Shoka tribe of Uttaranchal	Flesh	Strength, corpulence and virility good appetizer and alleviator of fatigue	The meat is believed to be a promoter of strength, corpulence and virility, is a good appetizer and alleviator of fatigue.	Negi and palyal (2007)

Table 8- Animals and their products used in skin problems in India.

**S. No.**		**English Name**	***Scientific Name***	**Tribe/ Inhabitant/ Region**	**Parts uses**	**Disease**	**Method of preparation and medicinal use**	**Related reported author in [illegible]**
1.	Invertebrate	Honey bee	*Apis cerana indica (Fabricius 1798)*	Tirunelveli district, Tamil Nadu	Honey	Skin abrasions,	Applied externally.	Ranjit Singh et al (2004)
2.	Invertebrate	Honey bee	*Apis cerana* Apis dorsata	Tirunelveli district, Tamil Nadu	Honey	Skin abrasions,	Applied externally.	Ranjit Singh et al (2004)

			(Fabricius, 1793)					
3.	Invertebrate	Honey bee	Apis florea (Fabricius, 1787)	Tirunelveli district, Tamil Nadu	Honey	Skin abrasions,	Applied externally.	Ranjit Singh et al (2004)
4.	Invertebrate	Pod Borer	*Helicoverpa armigera (Hubner, 1805)*	Chhattishgadh	Caterpillar	Hair growth	Aqueous extracts of caterpillar to promote hair growth.	Oodhia (1995)
5.	Invertebrate	Housefly	*Musca domestica nebulo (Fabricius. 1784)*	Chhattishgadh	Whole body	Dyeing the hair black	100 houseflies and mix them in sesamum oil. After mixing, the oil is kept under open sky in direct sunlight for forty days. Them this oil is used to dye the hair black.	Oodhia (1995)
6.	Invertebrate	Green Leafhoppe r (GLH)	*Nephotettix nigropictus (Stal, 1870)*	Chhattishgadh	Whole body	Skin problems		Oodhia (1995)
7.	Reptile	Earth Boa	*Eryx johnii (Russell, 1801)*	Nandurbar district of Maharashtra	Whole body	Leucoderma	The oil of earth boa is applied to treat leucoderma.	Patil (2003)
8.	Reptile	python	*Python reticulatus (Schneider, 1801)*	Kanikaran, Kattunaikkan and Toda	Fat	Leprosy	The fat deposits are carefully collected from the gonads and melted in an earthen pot and applied on the affected.	Solavan et al (2004)
9.	Aves	Pigeon	*Columba livia (Gmelin, 1789)*	Chhattishgadh	Excreta	Skin free from spots and to improve the fairness	Aqueous paste of excreta is best to make the skin free from spots and to improve the fairness.	Oodhia (2003 b)
10.	Mammal	Elephant	Elephas maximus indicus (Cuvier, 1798)	Tamilnadu tribes	tooth and tusk	Pimples	Scratched tooth and tusks are mixed with mother's milk and applied topically on the affected part before going to bed.	Solavan et al (2004)
11.	Mammal	Mithun	*Bos gaurus (H. Smith, 1827)* Synonym- *Bos frontalis*	Ao, Angami, Sema, Khiamniungan, Chakhesang, Lotha, Zeliang, Sangtam, Yimchunger, Pochury, and Rengma tribes of Nagaland.	Flesh	Skin diseases	Cooked flesh is applied to cure skin diseases.	Jamir and Lal (2005)
12.	Mammal	Goat	*Capra sibirica* (Pallas, 1776)	Ao, Angami, Sema, Khiamniungan, Lotha, Chakhesang, Zeliang, Sangtam,	Urine	Skin disease	Drunk directly.	Kakati et al (2006), Jamir and Lal (2005)

				Pochury, Konyak and Rengma tribes of Nagaland.				
13.	Mammal	Sambhar	Cervus unicolor (Kerr, 1792)	Saharia tribe	Antler	Herpes	Antler is rubbed with water this paste is applied in Herpes.	Mahawar and Jaroli (2007)
14.	Mammal	Elephant	Elephas maximus indicus (Cuvier, 1798)	Ao, Angami, Sema, Khiamniungan, Chakhesang, Lotha, Zeliang, Sangtam, Pochury, Konyak and Rengma tribes of Nagaland.	Teeth and tusks	Leucoderma, Itching, eczema and ringworm infections	Teeth and tusks are powdered and applied on skin to treat leucoderma, eczema and ringworm infections.	Jamir and Lal (2005), Kakati et al (2006)
15.	Mammal	Human	*Homo sapiens (Linnaeus, 1758)*	Saharia tribe	Bones	Herpes	Bone is grounded with water and this paste is applied in Herpes.	Mahawar and Jaroli (2007)
16.	Mammal	Indian porcupine	*Hystrix indica (Kerr, 1792)*	Bhil, Garasia, Kathodia of Rajasthan	Quill	Leucoderma Scabies	A paste of quill and bone is prepare in water and applied on the white scars.	Sharma (2002)
17.	Mammal	Indian porcupine	*Hystrix indica (Kerr, 1792)*	Bhil, Garasia, Kathodia of Rajasthan	Bone	Leucoderma Scabies	A paste of quill and bone is prepare in water and applied on the white scars.	Sharma (2002)
18.	Mammal	Tiger	Panthera tigris (Linnaeus, 1758)	Kurimbas	Flesh	Leprosy	The fat melted in an earthen pot and applied on the affected parts.	Solavan et al (2004)
19.	Mammal	Tiger	Panthera tigris (Linnaeus, 1758)	Kurimbas	Fat	Leprosy	The fat melted in an earthen pot and applied on the affected parts.	Solavan et al (2004)

Table 9- Animals and their products used in wound healing in India

**S. No.**		**English Name**	***Scientific Name***	**Tribe/ Inhabitant/ Region**	**Parts uses**	**Disease**	**Method of preparation and medicinal use**	**Related reported author in [illegible]**
1.	Invertebrate	Honey bee	*Apis cerana indica (Fabricius 1798)*	Tirunelveli district, Tamil Nadu	honey	Small cuts, ulcers, mouth ulcers and wounds.	For throat infection or any inflammation, honey mixed with lime (calcium carbonate) is applied on the affected area.	Ranjit Singh et al (2004)
2.	Invertebrate	Honey bee	Apis dorsata (Fabricius, 1793)	Tirunelveli district, Tamil Nadu	honey	Small cuts, ulcers, mouth ulcers and wounds.	For throat infection or any inflammation, honey mixed with lime (calcium carbonate) is applied on the affected area.	Ranjit Singh et al (2004)

3.	Invertebrate	Honey bee	Apis florea (Fabricius, 1787)	Tirunelveli district, Tamil Nadu	honey	Small cuts, ulcers, mouth ulcers and wounds.	For throat infection or any inflammation, honey mixed with lime (calcium carbonate) is applied on the affected area.	Ranjit Singh et al (2004)
4.	Pisces	Fish	Schizothorax richardsonii (Gray, 1832)	Shoka tribe of Uttaranchal	Blood	Wounds	Fish Blood of cut fish is used in foot and mouth disease; it is also applied on sore wounds in legs too.	Negi and palyal (2007)
5.	Pisces	Fish	*Tor putitora (Hamilton, 1822)*	Shoka tribe of Uttaranchal	Blood	Wounds	Fish Blood of cut fish is used in foot and mouth disease; it is also applied on sore wounds in legs too.	Negi and palyal (2007)
6.	Reptile	Garden lizard	Calotes versicolor (Fitzinger, 1826)	Shoka tribe of Uttaranchal	Whole body	Wounds	The whole animal is boiled in oil and the oil is then used to heal wounds on cattle's body.	Negi and palyal (2007)
7.	Aves	Martens bird	*Martes flavigula (Boddaert, 1785)*	Shoka tribe of Uttaranchal	Bones	Wounds	A paste prepared from the bones of this bird is said to cure wounds.	Negi and palyal (2007)
8.	Mammal	Dog	Canis lupus familiaris (Linnaeus, 1758) Synonym- Canis familiaris	Kachchh	Flesh	Wounds	Excreta are applied and flesh is eaten to treat wounds.	Gupta Leena et al (2003)
9.	Mammal	Human	*Homo sapiens (Linnaeus, 1758)*	Ao, Angami, Sema, Khiamniungan, Chakhesang, Lotha, Zeliang, Sangtam, Pochury, Konyak and Rengma tribes of Nagaland., Mogya, Bawaria, Meena and Saharia tribe of Rajasthan	Urine	Wound healing	Urine is applied on eye injuries. Also applied as antiseptic on fresh wounds.	Jamir and Lal (2005), Mahawar and Jaroli (2006) (2007)
10.<br><br>11.	Mammal<br><br>Mammal	Porcupine<br><br>Rufous-tailed hare	*Hystrix indica (Kerr, 1792)*<br><br>*Lepus nigricollis (F. Cuvier, 1823)*	Ao, Angami, Sema, Khiamniungan, Lotha, Sangtam and Rengma tribes of Nagaland.<br>Kachchh	Fat<br><br>Blood	Burn-wounds pain and rapid healing of fractured bones<br>Swelling	Fat is applied to relieve burn-wounds pain and rapid healing of fractured bones.<br><br>Blood is applied externally for healing swelling	Jamir and Lal (2005)<br><br>Gupta Leena et al (2003)

Table 10- Animals and their products used in eye and ears problems in India.

S. No.		English Name	*Scientific Name*	Tribe/ Inhabitant/ Region	Parts uses	Disease	Method of preparation and medicinal use	Related reported author in India
1.	Invertebrate	Lightening Beetles or Fireflies or Lighting bugs	Photuris lucicrescens (Barber, 1951)	Chhattishgadh	Whole body	Cold borne earache		Oodhia (1995)
2.	Invertebrate	Honey bee	*Apis cerana indica (Fabricius 1798)*	Mogya, Bawaria, Meena and Saharia tribe of Rajasthan	Honey	Eye disease	Used as eye drops to cure eye disease.	Mahawar and Jaroli (2006) (2007)
3.	Invertebrate	Apple Snail	Pila globosa (Swainson, 1822)	Ao, Angami, Sema, Khiamniungan, Chakhesang, Lotha, Sangtam, Pochury, Konyak and Rengma tribes of Nagaland.	Flesh	Eye related problems.	Flesh is eaten after cooking as eye related problems.	Jamir and Lal (2005)
4.	Invertebrate	Hermit Crab	*Uca pugnax*	Kachchh	Whole body	Ear pains	Crab is boiled in oil and used externally for curing ear pains.	Gupta Leena et al (2003)
5.	Reptile	Snakes	Gloydius himalayanus (Günther, 1864) Synonym- *Ancistrodon himalayans*	Shoka tribe of Uttaranchal	Flesh	Eyesight,	The meat is believed to promote eyesight.	Negi and palyal (2007)
6.	Reptile	Snakes	*Ptyas mucosus (Linnaeus, 1758)*	Shoka tribe of Uttaranchal	Flesh	Eyesight,	The meat is believed to promote eyesight.	Negi and palyal (2007)
7.	Reptile	Snakes	Daboia russelii (Shaw & Nodder, 1797) Synonym- *Vipera russelli*	Shoka tribe of Uttaranchal	Flesh	Eyesight,	The meat is believed to promote eyesight.	Negi and palyal (2007)
8.	Aves	Crow-pheasant	*Centropus sinensis (Stephens, 1815)*	Ao, Angami, Sema, Khiamniungan, Chakhesang, Lotha, Zeliang, Sangtam, Pochury, Konyak and Rengma tribes of	Bone	Earache	Bone is crushed to powder and suspended in water to prepare ear drop to relieve earache.	Jamir and Lal (2005)

				Nagaland.				
9.	Aves	Indian Roller	*Coracias benghalensis (Linnaeus, 1758)*	Bhil, Garasia, Kathodia of Rajasthan	Feather	Earache and pus in ear	Feathers are rubbed in water and the ascence is used as an eardrop.	Sharma (2002)
10.	Aves	Crow	Corvus splendens (Vieillot, 1817)	Ao Tribe of Nagaland	Bones	Earache	Crushed into powder, mixed with water and applied in ear drop by drop.	Kakati et al (2006)
11.	Aves	Horse	*Equus caballus (Linnaeus, 1758)*	Shoka tribe of Uttaranchal	Flesh	Eyesight.	Meat is believed to be a promoter of eyesight.	Negi and palyal (2007)
12.	Aves	Indian Peafowl Or Peacock	*Pavo cristatus (Linnaeus, 1758)*	Mogya, Bawaria, Meena and Saharia tribe of Rajasthan Nandurbar district of Maharashtra Bhil, Garasia, Kathodia of Rajasthan, Kachchh Ao, Angami, Sema, Khiamniungan, Lotha, Zeliang, Konyak tribes of Nagaland.,	Leg	Ear infections	Peacock's leg is rubbed with water and this essenced water is used in ear infections. Some tribes are boiled with oil, which is used to treat the ear pain.	Mahawar and Jaroli (2006), (2007) Patil (2003), Sharma (2002), Gupta Leena et al (2003), Jamir and Lal (2005), Kakati et al (2006)
13.	Mammal	Cattle	Bos taurus (Linnaeus, 1758) *Synonym- Bos indicus*	Shoka tribe of Uttaranchal	Bile	Eye diseases	It cures eye diseases.	Negi and palyal (2007)
14.	Mammal	Dog	Canis lupus familiaris (Linnaeus, 1758) Synonym- Canis familiaris	Ao, Angami, Sema, Khiamniungan, Chakhesang, Lotha, Zeliang, Sangtam, Yimchunger, Pochury, Konyak and Rengma tribes of Nagaland., Mogya, Bawaria, Meena tribe of Rajasthan	Urine	Ear Problem	Used as eardrop for curing earache.	Mahawar and Jaroli (2006), Jamir and Lal (2005)
15.	Mammal	Goat	*Capra sibirica (Pallas, 1776)*	Ao, Angami, Sema, Khiamniungan, Chakhesang, Lotha, Zeliang, Sangtam, Pochury, Konyak and Rengma tribes of Nagaland.	Urine	Night blindness,	Urine is taken to cure night blindness.	Jamir and Lal (2005)

16.	Mammal	Sambhar	Cervus unicolor (Kerr, 1792)	Mogya, Bawaria, Meena tribe of Rajasthan and Kachchh	Antler	Eye ailments	Antler is rubbed with water this paste is applied in eye ailments.	Mahawar and Jaroli (2006), Gupta Leena et al (2003)
17.	Mammal	elephant	Elephas maximus indicus (Cuvier, 1798)	Tamilnadu tribes	tooth and tusk	Conjunctivitis	Scratched tooth and tusks are mixed with mother's milk and applied topically on the affected part before going to bed.	Solavan et al (2004)
18.	Mammal	Human	*Homo sapiens (Linnaeus, 1758)*	Ao, Angami, Sema, Khiamniungan, Chakhesang, Lotha, Zeliang, Sangtam, Pochury, Konyak and Rengma tribes of Nagaland.	Milk	Eyeache	Milk is applied as eye drop to relieve eyeache.	Jamir and Lal (2005)
19.	Mammal	Bat	*Myotis lucifugus (LeConte, 1831)*	Ao, Angami, Sema, Khiamniungan, Chakhesang, Lotha, Zeliang, Pochury, Konyak and Rengma tribes of Nagaland.	Flesh	Night blindness	Flesh is cooked and eaten for curing night blindness.	Jamir and Lal (2005)
20.	Mammal	Tiger	*Panthera tigris (Linnaeus, 1758)*	Ao, Angami, Sema, Khiamniungan, Chakhesang, Lotha, Zeliang, Sangtam, Pochury, Konyak and Rengma tribes of Nagaland.	Milk	Cataract and night blindness	Its milk cures cataract and night blindness.	Jamir and Lal (2005)

Table 11 - Animals and their products used in burn in India.

**S. No.**		**English Name**	***Scientific Name***	**Tribe/ Inhabitant/ Region**	**Parts uses**	**Disease**	**Method of preparation and medicinal use**	**Related reported author in India**
1.	Invertebrate	Velvet ant	*Dasymutilla occidentalis (Linnaeus)*	Tirunelveli district, Tamil Nadu	Whole body	Inflammation	Along with bees wax, the powder of this ant is used to prepare ointment to treat inflammation.	Ranjit Singh et al (2004)

2.	Amphibia	Frog	Fejervarya limnocharis synonym- *Lymnonecties limnorcharis*	Ao, Angami, Sema, Khiamniungan, Chakhesang, Lotha, Zeliang, Sangtam, Pochury, Konyak and Rengma tribes of Nagaland.	Skin	Burn wounds	Skin is applied on burn wounds for rapid healing.	Jamir and Lal (2005)
3.	Amphibia	Frog	Hoplobatrachus tigerinus (Daudin, 1803) *synonym-Rana tigrina*	Shoka tribe of Uttaranchal	Whole body	Burn	Frog boiled in oil is used for healing wounds due to burn.	Negi and palyal (2007)
4.	Reptile	Hardshelled Turtle.	*Kachuga tentoria (Gray, 1834)*	Saharia tribe	Carapace	Burn	Ash of carapace mix with coconut oil and use for skin burns.	Mahawar and Jaroli (2007)
5.	Reptile	Monitor	*Varanus bengalensis (Daudin, 1758)*	Ao Tribe of Nagaland Nandurbar district of Maharashtra	Fat	Burn	The fat (oil) of this lizard is used for Burn.	Kakati et al (2006), Patil (2003)
6.	Aves	Eagle	*Aquila rapax (Temminck, 1828)*	Ao Tribe of Nagaland	Fat	Burn	Applied locally.	Kakati et al (2006)
7.	Aves	Domestic fowl	*Gallus gallus domesticus*	Ao Tribe of Nagaland	Fat	Burn	Raw fat warmed and applied locally.	Kakati et al (2006)
8.	Mammal	Rufous-tailed hare	*Lepus nigricollis (F. Cuvier, 1823)*	Kachchh	Tail	Burning sensation	The ash of tail is mixed with oil and the prepared paste is applied for curing burning sensation.	Gupta Leena et al (2003)
9.	Mammal	Black ibis	*Pseudibis papillosa (Temminck, 1824)*	Kachchh	Blood	Burning sensation,	Blood is massaged for curing burning sensation.	Gupta Leena et al (2003)
10.	Mammal	Domesticated pig	Sus scrofa *domestica*	Ao Tribe of Nagaland	Fat	Burn	Applied locally as embrocation/ Massager and in snakebite Used as antidote.	Kakati et al (2006)

Table 12- Animals and their products used in antidotes in India.

S. No.		English Name	*Scientific Name*	Tribe/ Inhabitant/ Region	Parts uses	Disease	Method of preparation and medicinal use	Related reported author in
1.	Invertebrate	Earthworm	*Pheretima posthuma (L. Vaillant, 1868)*	Ao, Angami, Sema, Khiamniungan, Chakhesang, Lotha, Zeliang, Sangtam, Pochury, Konyak and Rengma tribes of Nagaland.	Whole body	Snake and spider bites	Earthworm is eaten raw to serve as an antidote in snake and spider bites.	Jamir and Lal (2005)
2.	Aves	Python	Python reticulatus (Schneider, 1801)	Ao, Angami, Sema, Khiamniungan, Chakhesang, Zeliang, Sangtam, Pochury, Konyak and Rengma tribes of Nagaland.	Bile duct	Spider and snake bite	Its bile duct is used as an antidote against spider and snake bite.	Jamir and Lal (2005)
3.	Aves	Monitor	*Varanus bengalensis (Daudin, 1758)*	Ao Tribe of Nagaland	Bile duct	Spider and snake bite	Taken orally as antidote.	Kakati et al (2006)
**4.**	Mammal	Cow	Bos taurus (Linnaeus, 1758) *Synonym- Bos indicus*	Mogya, Bawaria, Meena tribe of Rajasthan	Ghee	Snake poison	250gm Ghee + 100gm Black pepper mixture given orally to neutralize snake poison.	Mahawar and Jaroli (2006)
5.	Mammal	Cattle	Bos taurus (Linnaeus, 1758) *Synonym- Bos indicus*	Shoka tribe of Uttaranchal	Bile	Poisoning,	It cures poisoning.	Negi and palyal (2007)
6.	Mammal	Flying squirrel	*Petaurista petaurista (Pallas, 1766)*	Ao Tribe of Nagaland	Intestine	Anti poison	Cooked and eaten.	Kakati et al (2006)
7.	Mammal	Domesticated pig	Sus scrofa *domestica*	Ao Tribe of Nagaland	Fat	Snake bite	Applied locally as embrocation/ massager and in snakebite used as antidote.	Kakati et al (2006)

Table 13- Animals and their products used in gynecological problems in India.

S.N.		English Name	*Scientific Name*	Tribe/ Inhabitant/ Region	Parts uses	Disease	Method of preparation and medicinal use	Related reported author in [illegible]

**1.**	Invertebrate	Silkworm	*Bombyx mori (Linnaeus)*	Tirunelveli district, Tamil Nadu	Cocoon	Menstruation problem, leucorrhoea	The cocoon ash of the silkworm is used as a styptic, tonic and astringent to check profuse menstruation, leucorrhoea and chronic diarrhea.	Ranjit Singh et al (2004)
2.	Invertebrate	Green Leafhopper (GLH)	*Nephotettix nigropictus (Stal, 1870)*	Chhattishgadh	Whole body	Gonorrhea		Oodhia (1995)
3.	Aves	Pigeon	*Columba livia (Gmelin, 1789)*	Chhattishgadh	Excreta	Pregnancy	Putting the dried excreta inside the vagina of childless woman is very effective to make her pregnant.	Oodhia (2003 b)
4.	Mammal	Himalayan Thar	*Hemitragus jemlahicus (H. Smith, 1826)*	Shoka tribe of Uttaranchal	Soup of the bones	Parturition and lactating	The soup of the bones (of the limbs) is principally given to the womenfolk immediately after giving birth to a child, and usually during the parturition and lactating months.	Negi and palyal (2007)
5.	Mammal	Hare	Oryctolagus cuniculus (Linnaeus, 1758)	Shoka tribe of Uttaranchal	Flesh	Menstrual disorders	The meat is believed to cure menstrual disorders.	Negi and palyal (2007)
6.	Mammal	Hanuman Monkey	Semnopithecus entellus (Dufresne, 1797) Synomyme- *Presbytis entellus*	Kathodia of Rajasthan	Flesh	Facilitates delivery	Flesh is eaten by women at the time of child birth.	Sharma (2002)

Table 14- Animals and their products used in miscellaneous diseases in India.

**S.N.**		**English Name**	***Scientific Name***	**Tribe/ Inhabitant/ Region**	**Parts uses**	**Disease**	**Method of preparation and medicinal use**	**Related reported author in India**
1.	Invertebrate	Lightening Beetles or Fireflies or Lighting bugs	Photuris lucicrescens (Barber, 1951)	Chattishgadh	Whole body	Styptic to stop the bleeding		Oodhia (1995)
2.	Invertebrate	Bedbugs	*Cimex rotundatus (Signoret, 1852)*	Shoka tribe of Uttaranchal	Whole body	Ringworm	Bedbugs crushed in holy basil- Ocimum sanctum is applied on ringworm.	Negi and palyal (2007)
3.	Invertebrate	Pod Borer	*Helicoverpa armigera*	Chattishgadh	Caterpillar	Eosinophilia	Helicoverpa caterpillar feeding on	Oodhia (1995)

			(Hubner, 1805)				Sphaeranthus leaves is used for the treatment of eosinophilia and asthma.	
4.	Invertebrate	Housefly	*Musca domestica nebulo (Fabricius. 1784)*	Chattishgadh	Whole body	To cure the cold fever	One housefly, half black pepper (Piper longum) and one pinch of asafoetida are mixed and this mixture is applied on eyes.	Oodhia (1995)
5.	Invertebrate	Weaver ant	*Oecophylla smaragdina (Fabricius, 1775)*	Tirunelveli district, Tamil Nadu	Eggs	Tetanic fever, high fever and even malarial fever	The eggs of this insect are used to prepare medicine with several species of medicinal herbs and administered to patients suffering from tetanic fever, ear pain, high fever and even malarial fever.	Ranjit Singh et al (2004)
6.	Invertebrate	Red Velvet Mite	*Trombidium grandissimum (Koch, 1867)*	Chattishgadh	Whole body	Malaria		Oodhia (1995)
7.	Pisces	Carp fish	*Labeo gonius (Hamilton, 1822)*	Dibrugarh District, Assam	Edible portion of the fish	Obesity	Curry prepared from the edible portion of fish is taken with rice on Saturday. The same process is repeated for another eight consecutive Saturdays.	Kalita (2005)
8.	Pisces	Cuchia eel	*Monopterus cuchia (Hamilton, 1822)*	Dibrugarh District, Assam	Blood	Diabetes	Fresh blood of a *M. cuchia* is taken orally empty stomach in the morning for four days. After completion of the above dosage an overnight dry *L. aspera* plant extract (two teaspoon) mixed with one spoonful of honey is allowed to lick mixed with one spoonful of honey. After 20 minutes about one-fourth glass of lukewarm water is given to drink and the process is continued for another two days.	Kalita (2005)
9.	Reptile	Common Garden Lizard	*Calotes versicolor (Fitzinger, 1826)*	Bhil, Garasia, Kathodia of Rajasthan	Flesh	Injuries of toes	Warm flesh is kept on injuries of toes caused by stumbling.	Sharma (2002)
10.	Reptile	Indian Flap shell turtle	*Lissemys punctata (Lacépède, 1788)*	Kachchh	Carapace	Internal injuries, pruritis,	Carapace is burnt and ash is used for healing of internal injuries, pruritis.	Gupta Leena et al (2003)
11.	Aves	Jangli Murga (Wild Cock)	*Gallus sonneratii (Temminck, 1813)*	Chattishgadh	Gall bladder	Diabetes	The patients to swallow the Pitta (Gall bladder) regularly up to seven days to get significant	Oodhia (2003 a)

							results.	
12.	Aves	Pigeon	*Columba livia (Gmelin, 1789)*	Chattishgadh	Excreta	Carbuncle	The patients to swallow the small dose of excreta with gud (jaggery).	Oodhia (2003 b)
13.	Aves	Pigeon	*Columba livia (Gmelin, 1789)*	Chattishgadh	Excreta	Enuresis (bed wetting)	Swallow fresh excreta with gud (Jaggery) utpto one week.	Oodhia (2003 b)
14.	Aves	Hen	*Gallus gallus domesticus*	all the Thailand tribes	Egg albumin	Haematoma	Five gram of the solid leaf pulp of *Aloe littoralis* is soaked in egg albumin for 3 hrs.	Solavan et al (2004)
15.	Aves	House sparrow	*Passer domesticus (Linnaeus, 1758)*	Ao Tribe of Nagaland	Flesh	Stammering	Boiled in water and taken.	Kakati et al (2006)
16.	Aves	Peacock	*Pavo cristatus (Linnaeus, 1758)*	Except Kanikaran, all the Tamilnadu tribes	Flesh	Contracted limbs	The fat content of the visceral organs is melted over a short flame and applied externally on the contracted limbs.	Solavan et al (2004)
17.	Aves	Peacock	*Pavo cristatus (Linnaeus, 1758)*	Except Kanikaran, all the Tamilnadu tribes	Fat	Contracted limbs	The fat content of the visceral organs is melted over a short flame and applied externally on the contracted limbs.	Solavan et al (2004)
18.	Aves	Peacock	*Pavo cristatus (Linnaeus, 1758)*	Except Kanikaran, all the Tamilnadu tribes	Feather	Hiccups	Ten gram each of the seeds of *Piper longum* and *Cuminum cyminum* are powdered and mixed with 10 g ash of peacock's feather.	Solavan et al (2004)
19.	Mammal	Camel	Camelus dromedarius (Linnaeus, 1758)	Chhattishgadh	Excreta	Hydrocele	Excreta with haldi (Curcuma longa) and ginger (Zingiber officinalis) are a best remedy for hydrocele.	Oodhia (2003 b)
20.	Mammal	Cow	Bos taurus (Linnaeus, 1758) *Synonym- Bos indicus*	Mogya, Bawaria, Meena tribe of Rajasthan	Urine	Cancer	Given to cure cancer.	Mahawar and Jaroli (2006)
21.	Mammal	Sambhar	*Cervus unicolor (Kerr, 1792)*	lrular and Sholaga tribes	Penis	Hydrocele	The dried penis is scratched in water and it is applied on the scrotum of man till the symptoms of hydrocele disappear.	Solavan et al (2004)
22.	Mammal	Indian porcupine	*Hystrix indica (Kerr, 1792)*	Bhil, Garasia, Kathodia of Rajasthan	Fat	Internal tumours	Freshly killed porcupine is boiled with water to extract fat and it’s taken orally to cure internal tumours.	Sharma (2002)
23.	Mammal	Monkey	*Macaca mulatta (Zimmermann, 1780)*	Shoka tribe of Uttaranchal	Flesh	Adiposity, and parasitic infestation.	The meat is believed to cure, adiposity, anemia and parasitic infestation.	Negi and palyal (2007)

24.	Mammal	Indian Pangolin	*Manis crassicaudata (Gray, 1827)*	Bhil, Garasia, Kathodia of Rajasthan	Scale	General and pregnant women feet Swelling	A paste is prepared in water by rubbing the Scales on stone and applied.	Sharma (2002)
25.	Mammal	Musk deer	Moschus moschiferus (Linnaeus, 1758)	Shoka tribe of Uttaranchal	Musk	Immune system in children	The lactating mother are given a pinch of 'kasturi'-the musk with the belief that the children receiving the milk become immune to diseases, i.e. the musk is believed to bolster the immune system in children.	Negi and palyal (2007)
26.	Mammal	Musk deer	Moschus moschiferus (Linnaeus, 1758)	Shoka tribe of Uttaranchal	Musk	Malaria, high fever and in heart ailments	Musk is used in the treatment of malaria, high fever and in heart ailments.	Negi and palyal (2007)
27.	Mammal	Sherni (Tigress)	Panthera tigris (Linnaeus, 1758)	Chattishgadh	Excreta	Alcoholic drinks.	The healers give the addicted natives, a glass of drink (the last drink) and add few pinches of dried excreta in it.	Oodhia (2003 a)
28.	Mammal	Rat	*Rattus rattus (Linnaeus, 1758)*	Kachchh	Whole body	Bald head	The animal is roasted and ash is mixed with mustard oil. This paste is mixed with the crushed leaves of *Rivea hypocrateriformis* (Fungvel) and Flowers of *Azadirachta indica* (Neem) and on the bald head.	Gupta Leena et al (2003)
29.	Mammal	Rat	*Rattus rattus (Linnaeus, 1758)*	Chattishgadh	Excreta	Mad dog bite	The traditional healers give patients the mixture containing Bhavri insect and rat excreta with Gud (Juggery).	Oodhia (2003 b)
30.	Mammal	Himalayan black bear	Ursus thibetanus (G. Cuvier, 1823) Synonym- *Selenarctos thibetanus*	Shoka tribe of Uttaranchal	Gall bladder	Malaria	Its gall bladder is used for curing myriad diseases, malaria being one of them. The fresh gall bladder is filled with rice grains and then is left to dry out. After few days a yellowish powder is formed, which is used as medicine.	Negi and palyal (2007)
31.	Mammal	Pig	Sus scrofa *domestica*	all the tribes except Toda	Fat	Hemorrhoids	The fat content of the adipose tissue of the pig is melted in an earthen pot and applied topically until hemorrhoids disappear.	Solavan et al (2004)
